# Interference with plastome gene expression and Clp protease activity in Arabidopsis triggers a chloroplast unfolded protein response to restore protein homeostasis

**DOI:** 10.1371/journal.pgen.1007022

**Published:** 2017-09-22

**Authors:** Ernesto Llamas, Pablo Pulido, Manuel Rodriguez-Concepcion

**Affiliations:** Centre for Research in Agricultural Genomics (CRAG) CSIC-IRTA-UAB-UB, Campus UAB Bellaterra, Barcelona, Spain; University of Massachusetts Medical School, UNITED STATES

## Abstract

Disruption of protein homeostasis in chloroplasts impairs the correct functioning of essential metabolic pathways, including the methylerythritol 4-phosphate (MEP) pathway for the production of plastidial isoprenoids involved in photosynthesis and growth. We previously found that misfolded and aggregated forms of the first enzyme of the MEP pathway are degraded by the Clp protease with the involvement of Hsp70 and Hsp100/ClpC1 chaperones in *Arabidopsis thaliana*. By contrast, the combined unfolding and disaggregating actions of Hsp70 and Hsp100/ClpB3 chaperones allow solubilization and hence reactivation of the enzyme. The repair pathway is promoted when the levels of ClpB3 proteins increase upon reduction of Clp protease activity in mutants or wild-type plants treated with the chloroplast protein synthesis inhibitor lincomycin (LIN). Here we show that LIN treatment rapidly increases the levels of aggregated proteins in the chloroplast, unleashing a specific retrograde signaling pathway that up-regulates expression of *ClpB3* and other nuclear genes encoding plastidial chaperones. As a consequence, folding capacity is increased to restore protein homeostasis. This sort of chloroplast unfolded protein response (cpUPR) mechanism appears to be mediated by the heat shock transcription factor HsfA2. Expression of *HsfA2* and cpUPR-related target genes is independent of GUN1, a central integrator of retrograde signaling pathways. However, double mutants defective in both GUN1 and plastome gene expression (or Clp protease activity) are seedling lethal, confirming that the GUN1 protein is essential for protein homeostasis in chloroplasts.

## Author summary

Chloroplasts are central metabolic factories for plant cells. Yet, they are constantly challenged by stress episodes that alter protein homeostasis and disrupt normal chloroplast functions. To deal with this problem, protein quality control pathways involving particular chaperones and proteases promote correct protein folding and remove irreversibly damaged proteins. In the case of DXS, the main regulatory enzyme of the isoprenoid pathway, misfolded and aggregated forms of the enzyme are refolded back to its active form by stromal chaperones of the Hsp70 and Hsp100/ClpB families, hence preventing their degradation by the Clp protease complex. In this paper we report that saturated or defective Clp protease activity triggers a chloroplast unfolded protein response that results in the up-regulation of nuclear genes encoding chloroplast chaperones. Higher levels of these chaperones (particularly the disaggregase ClpB3) eventually restore the activity of DXS and other chloroplast proteins that accumulate in a non-functional form when Clp protease activity and chloroplast functions are compromised.

## Introduction

Endosymbiotic organelles such as mitochondria and chloroplasts play fundamental roles in eukaryotic organisms. They both contain their own genome but most of their proteins are encoded by the nuclear genome. As a consequence, mechanisms to adjust nuclear gene expression to particular organelle needs are required to ensure an appropriate supply of functional proteins [1–4]. Nuclear-encoded proteins are translocated into organelles in unfolded form, and then their transit peptide is cleaved before they are properly folded, assembled, or/and targeted to their particular suborganellar destination. Inside the organelles, the lifespan and activity of proteins depend on protein quality control (PQC) systems formed by chaperones and proteases that promote correct protein folding, prevent the formation of insoluble aggregates, and remove irreversibly damaged proteins. When misfolded proteins accumulate and aggregate in mitochondria, an adaptive transcriptional response known as unfolded protein response (UPR) is activated to communicate with the nucleus and induce the expression of nuclear genes encoding mitochondria-targeted chaperones and proteases [5–7]. The existence of a chloroplast UPR (cpUPR) has only recently been proposed based on work with the unicellular green alga *Chlamydomonas reinhardtii* [8,9]. In particular, gradual depletion of the catalytic capacity of the stromal Clp protease in algal cells was found to trigger the accumulation, both at the RNA and protein level, of small heat shock proteins, chaperones, and proteases [8]. *Arabidopsis thaliana* mutants with constitutively decreased Clp proteolytic activity also show highly increased levels of stromal chaperones from different families, including Cpn60, Hsp70, Hsp90, and Hsp100/ClpB [10–17]. Interestingly, the Clp protease is a key component of the UPR mechanism in mitochondria [6,18]. While these observations suggest that a UPR conceptually similar to that observed in mitochondria might operate in chloroplasts, the physiological signal(s) triggering this putative cpUPR and the specific consequences for chloroplast function remain unexplored.

**Fig 1:**
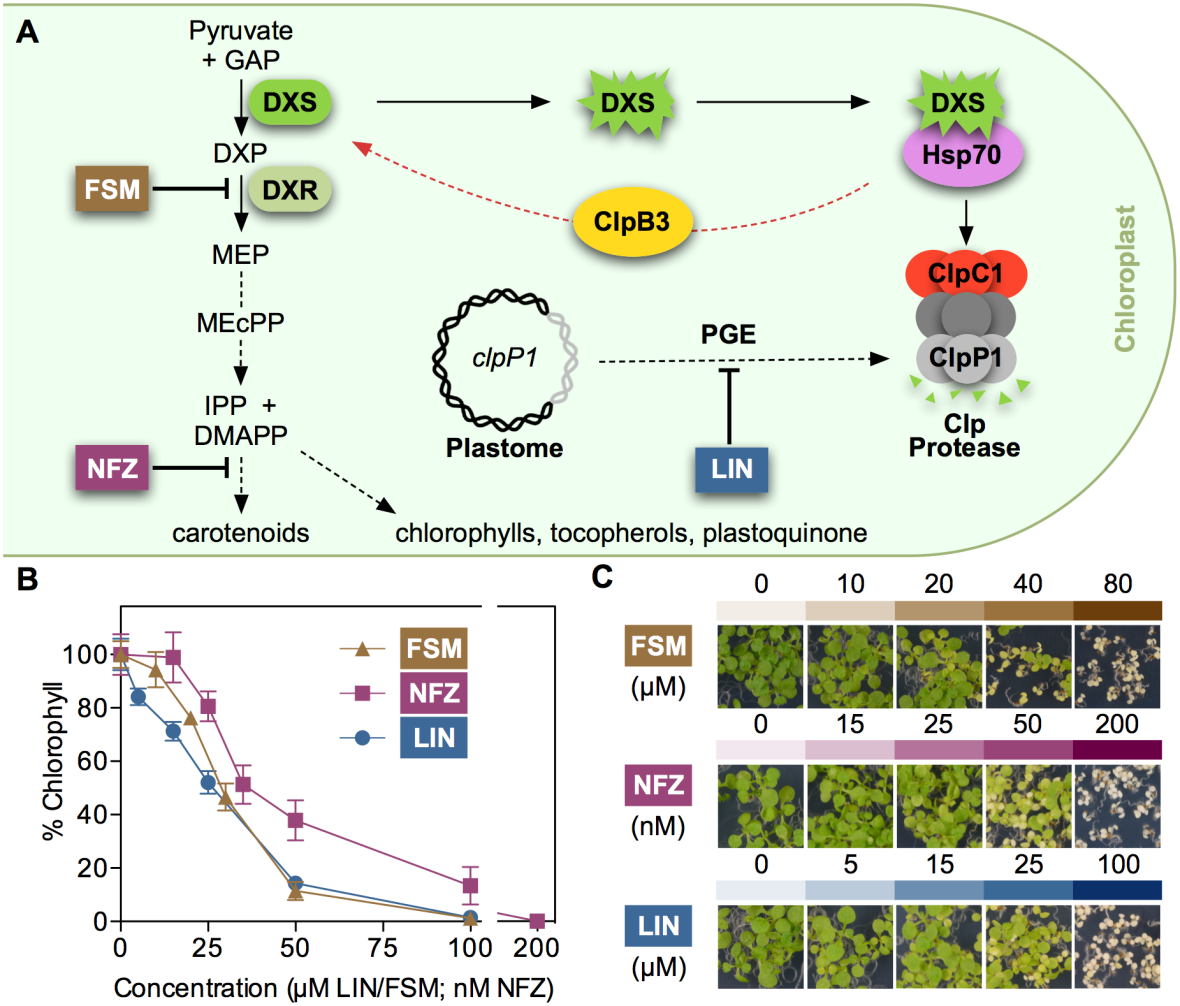
Inhibitors and mechanisms modulating metabolic flux to isoprenoids in chloroplasts. (A) Schematic representation of: (1) MEP pathway and derived products, with the position of enzymes (DXS, DXR) and inhibitor (FSM, NFZ) targets; (2) Hsp70-dependent pathways for misfolded and aggregated forms of DXS to be degraded (via ClpC1 and the Clp protease) or, alternatively, reactivated (via ClpB3); and (3) proposed mechanism by which interference with PGE (e.g. with LIN) impacts the activity of the Clp protease complex, based on the production of the plastome-encoded ClpP1 subunit. Red arrow represents the stress-induced refolding pathway. Dashed arrows represent multiple steps. GAP, glyceraldehyde 3-phosphate; DXP, deoxyxylulose 5-phosphate. See text for other acronyms. (B) Quantification of the inhibitor resistance phenotype estimated from chlorophyll levels in the absence (100%) or presence of inhibitors. (C) Representative images of Arabidopsis WT (Columbia) seedlings germinated and grown for 10 days under LD in the presence of the indicated concentrations of inhibitors.

Recently, we characterized the role of chloroplast PQC systems to control the levels and activity of Arabidopsis deoxyxylulose 5-phosphate synthase (DXS), the enzyme that catalyzes the first and main rate-determining step of the methylerythritol 4-phosphate (MEP) pathway [16,19,20]. The MEP pathway is localized in the plastid stroma and synthesizes the metabolic precursors for isoprenoids such as carotenoids and the prenyl chains of chlorophylls, tocopherols, or plastoquinone (Fig 1A). DXS is prone to misfold and aggregate, resulting in insolubility and loss of enzymatic activity [16,19,21]. Misfolded and aggregated forms of DXS are primarily degraded by the Clp protease complex through a pathway involving the DnaJ-like protein J20, an adaptor that delivers the inactive enzyme to stromal Hsp70 chaperones. Then, interaction with the Hsp100/ClpC1 chaperone allows unfolding of the DXS protein for degradation by the catalytic core of the complex. Alternatively, direct interaction of Hsp70 with Hsp100/ClpB3 eventually results in the refolding and hence reactivation of DXS (Fig 1A). ClpB3 is the only ClpB-type Hsp100 chaperone targeted to Arabidopsis plastids, where it is presumed to disaggregate protein clumps and promote protein solubilization either alone or in synergy with Hsp70 chaperones [22,23]. Unlike ClpB3, ClpC1 and the other two plastidial ClpC-type Hsp100 chaperones found in Arabidopsis (ClpC2 and ClpD) contain a ClpP-loop motif for interaction with proteolytic subunits of the Clp complex [16,22,24]. Notably, mutants defective in ClpC1 show an increase in ClpB3 protein levels that prevents the formation of DXS aggregates, eventually resulting in higher levels of enzymatically active DXS protein [16].

An enhanced accumulation of DXS protein levels (but not transcripts) was also observed upon genetic or pharmacological inhibition of plastome gene expression (PGE). Screening collections of Arabidopsis T-DNA insertion lines for plants able to survive in the presence of an otherwise lethal concentration of the MEP pathway inhibitor fosmidomycin (FSM) led to the isolation of *resistant to inhibition with FSM* (*rif*) mutants such as *rif10*, impaired in plastid RNA processing [25], and *rif1*, defective in plastidial ribosome assembly [26,27]. Besides DXS, both *rif10* and *rif1* mutants show enhanced accumulation of other MEP pathway enzymes, including deoxyxylulose 5-phosphate reductoisomerase (DXR), the specific target of FSM (Fig 1A). Accumulation of DXS and DXR enzymes resulting in enhanced FSM resistance was also observed when PGE was partially inhibited in wild-type (WT) seedlings grown in the presence of sublethal concentrations of chloramphenicol (CAP), an inhibitor of protein synthesis in plastids. By contrast, treatment with norflurazon (NFZ), an inhibitor of carotenoid biosynthesis (Fig 1A) that causes a similar visual phenotype without directly affecting PGE [28], did not result in FSM resistance [25]. As the catalytic ClpP1 subunit of the Clp protease is encoded by the plastome and its levels are altered in *rif* mutants and CAP-treated plants [26], it was proposed that interference with PGE disrupts stoichiometry and consequently reduces proteolytic activity of the Clp complex, eventually resulting in the accumulation of protein clients such as MEP pathway enzymes. The increase in ClpB3 protein levels observed when Clp activity is decreased would then contribute to maintain these enzymes in a correctly folded (i.e. active) form [16]. While increase of DXS and DXR protein levels in PGE-defective or Clp-impaired plants does not involve transcriptional changes [16,25,26], the mechanism up-regulating ClpB3 levels is currently unknown. In this work, we aimed to test the hypothesis that this mechanism might involve a cpUPR, i.e. that insufficient Clp protease activity resulting from PGE defects might elicit a retrograde signaling pathway eventually increasing the levels of plastidial chaperones (including ClpB3) by inducing the expression of the corresponding nuclear genes. Besides confirming this hypothesis and providing mechanistic insights, our results show that the plastidial protein GUN1 (a major integrator of signaling pathways from the chloroplast to the nucleus) is not required for the cpUPR-associated transcriptional changes but it is essential for chloroplast to respond to challenges disrupting protein homeostasis.

## Results

### Pharmacological and genetic interference with PGE triggers the accumulation of plastidial ClpB3 chaperones

**Fig 2:**
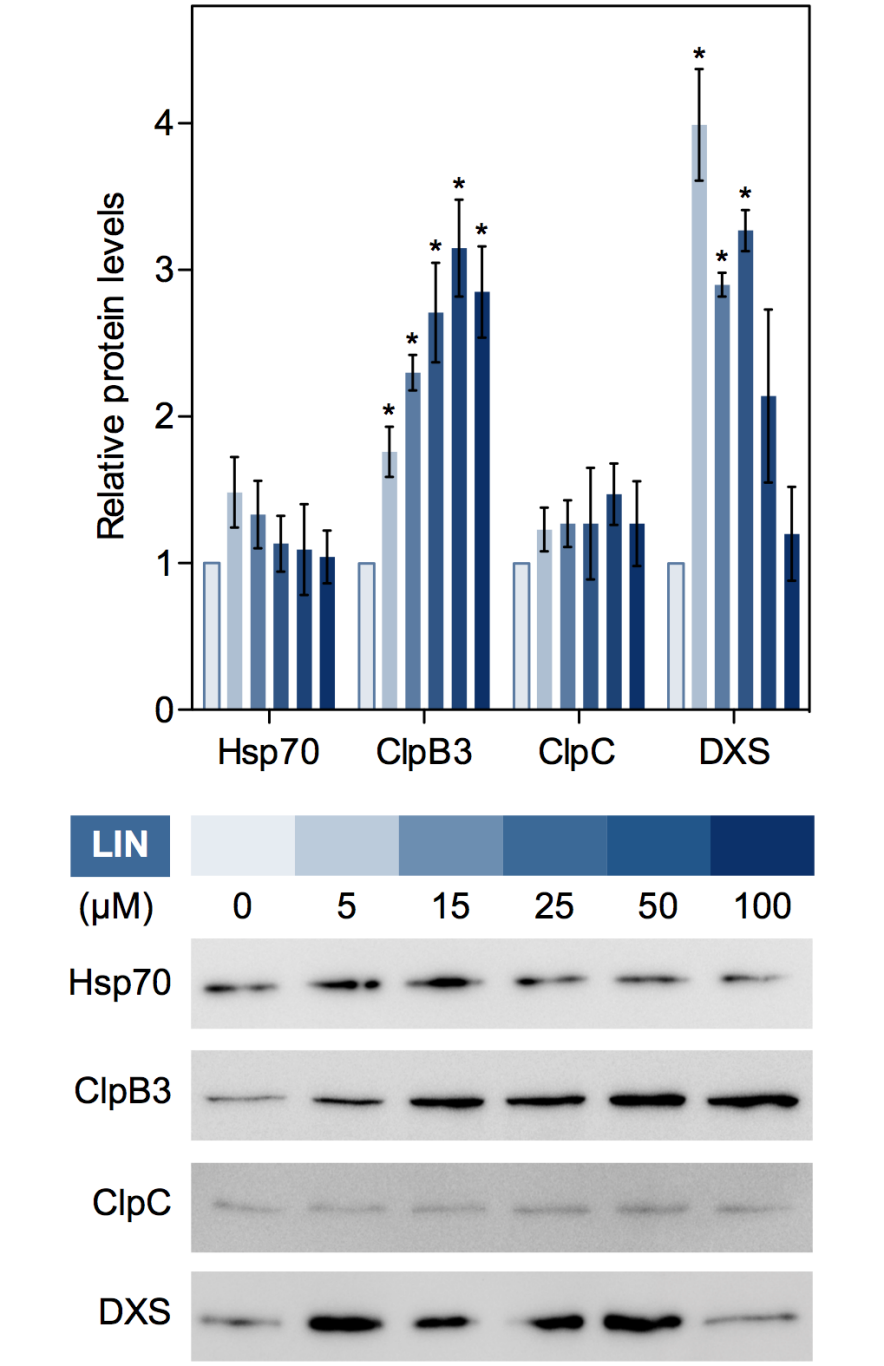
LIN treatment boosts accumulation of ClpB3 and DXS proteins. Chart represents Hsp70, ClpB3, ClpC and DXS protein levels detected by immunoblot analysis in 10-day-old WT plants grown at the concentrations of LIN indicated below. The mean and SEM values of n≥3 independent experiments are shown. Asterisks mark statistically significant differences (*t* test: p<0.05) relative to untreated controls. Representative immunoblots are also shown.

In mitochondria, UPR can be unleashed through interference with the expression of the organelle genome, e.g. by inhibiting protein translation [6]. To verify the possible existence of a cpUPR acting in response to alterations in PGE, we initially tested different concentrations of lincomycin (LIN) for their capacity to up-regulate the accumulation of plastidial chaperones. LIN specifically inhibits chloroplast translation even though it also has secondary effects on mitochondrial gene expression at high concentrations, i.e. those causing seedling bleaching [29,30]. WT (Columbia) Arabidopsis plants were germinated and grown for 10 days under long-day (LD) conditions in the presence of LIN at concentrations causing from no visible symptoms (5 μM) to complete bleaching (100 μM) (Fig 1B). Then, we analyzed the abundance of plastidial chaperones (Hsp70, ClpB3, and ClpC) by immunoblot analysis (Fig 2). While chaperones recognized by antibodies against plastidial Hsp70 and ClpC proteins hardly changed in LIN-exposed plants, the levels of ClpB3 did increase even at low concentrations of LIN (Fig 2). As concentration of LIN increased and plants became paler, levels of the ClpB3 unfoldase were progressively higher (Fig 2).

**Fig 3:**
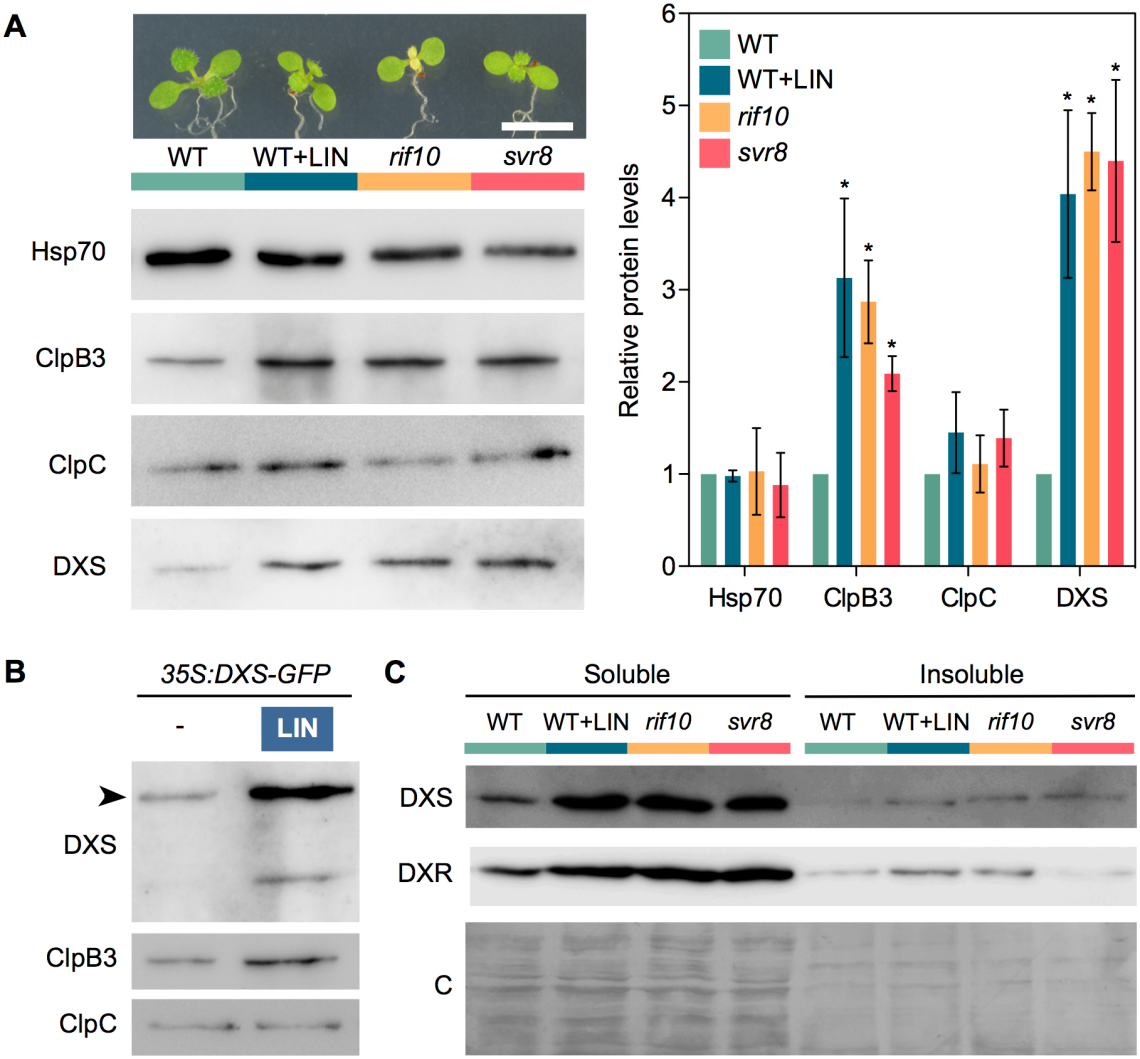
Interference with PGE promotes the accumulation of soluble DXS protein. (A) Immunoblot analysis of Hsp70, ClpB3, ClpC and DXS levels in WT plants and PGE-defective mutants *rif10-2* and *svr8-2*. PGE was also blocked in WT plants germinated and grown in the presence of 15 μM LIN (WT+LIN sample). Representative images of the plants used for immunoblot analysis are shown on top (bar, 5 mm). Graph shows the quantification of immunoblot data from n≥3 experiments represented as mean and SEM values relative to untreated WT plants. Asterisks mark statistically significant differences (*t* test: p<0.05) relative to WT samples. (B) Immunoblot analysis with the indicated antibodies of *35S*:*DXS-GFP* plants grown on media with (+) or without (-) 15 μM LIN. Arrowhead marks the position of the DXS-GFP protein. (C) DXS and DXR protein distribution in soluble and insoluble fractions isolated from the indicated samples. A Coomassie-Blue (C) staining of the blots is shown for reference.

We next used PGE-defective mutants to test whether ClpB3 accumulated to higher levels in their chloroplasts (Fig 3). In particular, we used mutants defective in the exoribonuclease RIF10, implicated in the processing of all major classes of plastid RNAs [25], or the chloroplast 50S ribosomal protein L24/SVR8 [31]. WT plants and T-DNA insertion alleles *rif10-2* and *svr8-2* were grown for 10 days under LD and then analyzed for chlorophyll and protein content. While both mutants showed similarly reduced chlorophyll levels (S1 Fig), *rif10-2* seedlings displayed green cotyledons and pale true leaves whereas *svr8-2* seedlings showed reduced pigmentation in both cotyledons and leaves (Fig 3A). WT plants growing on medium supplemented with 15 μM LIN, a concentration that reduced overall chlorophyll levels to those found in the mutants (S1 Fig), showed a general pale phenotype similar to the *svr8-2* mutant (Fig 3A). Similar to LIN-treated plants, levels of ClpB3, but not those of Hsp70 and ClpC chaperones, were increased in both *rif10-2* and *svr8-2* mutants compared to untreated WT controls (Fig 3A). Together, our data confirm that interference with PGE triggers the accumulation of particular chloroplast-targeted chaperones involved in releasing protein folding stress, such as ClpB3.

### LIN treatment also promotes accumulation of active MEP pathway enzymes and resistance to inhibitors of isoprenoid metabolism

**Fig 4:**
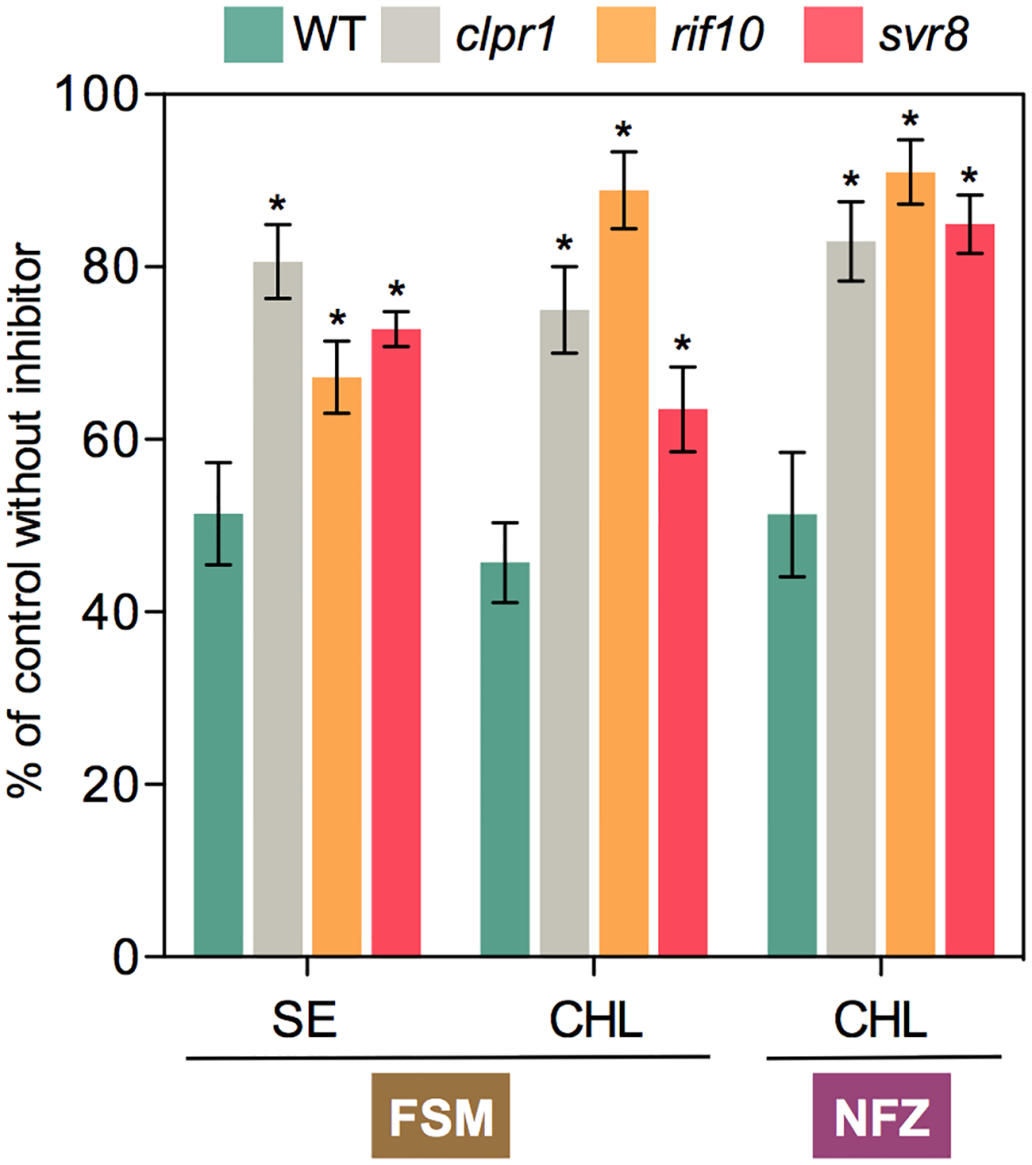
Mutants defective in PGE and Clp protease activity are resistant to plastidial isoprenoid inhibitors. Resistance to FSM was estimated by quantifying seedling establishment (SE, number of plants producing true leaves) and chlorophyll levels (CHL) in plants germinated and grown in the presence of 30 μM FSM relative to those obtained with no inhibitor (100%). Similarly, NFZ resistance was calculated based on chlorophyll levels in media with 35 nM NFZ. Data correspond to the mean and SEM values of n≥3 independent experiments and asterisks mark statistically significant differences (*t* test: p<0.05) relative to WT samples.

**Fig 5:**
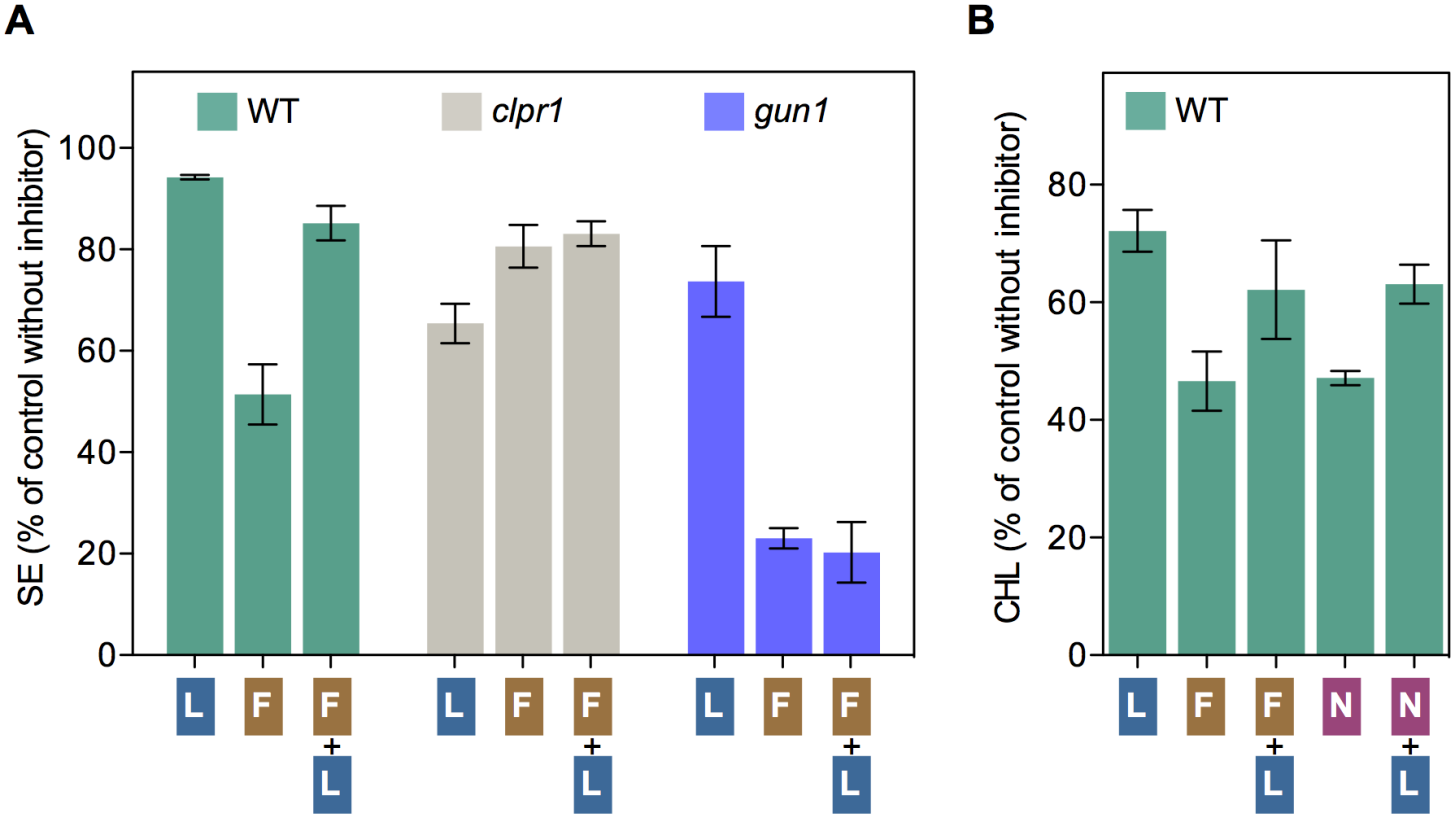
Resistance to FSM and NFZ is improved by disrupting PGE with LIN. (A) Resistance of WT plants and the indicated mutants was estimated by quantifying SE after germination and growth on media supplemented with 15 μM LIN (L), 30 μM FSM (F), or both (F+L) relative to non-supplemented medium. (B) Resistance of WT plants quantified as CHL levels in media supplemented with 15 μM LIN (L), 30 μM FSM (F), 35 nM NFZ (N) or the indicated combinations relative to non-supplemented medium. Data correspond to the mean and SEM values of n≥3 independent experiments.

ClpB3 helps to refold misfolded and aggregated forms of DXS to recover their solubility and enzymatic activity, hence preventing their Clp-mediated degradation [16] (Fig 1A). As a consequence, it was expected that up-regulation of ClpB3 upon interference with PGE would correlate with higher levels of soluble (i.e. enzymatically active) DXS. Indeed, DXS protein levels were increased in PGE-defective *rif10-2* and *svr8-2* mutants and LIN-treated WT plants (Figs 2 and 3A) whereas levels of DXS-encoding transcripts remained unchanged (S1 Fig). Transgenic *35S*:*DXS-GFP* lines constitutively expressing a GFP-tagged DXS protein [19] also showed enhanced levels of both endogenous DXS and recombinant DXS-GFP proteins when treated with 15 μM LIN (Fig 3B), consistent with the conclusion that DXS protein accumulation in plants with PGE defects does not rely on transcriptional changes. Furthermore, the vast majority of the DXS protein accumulated in plants with a genetically or pharmacologically impaired PGE remained in the soluble (stromal) fraction (Fig 3C). Similar to DXS, the next enzyme of the MEP pathway, DXR, was also found to be mostly soluble in untreated WT plants and to strongly accumulate in the soluble fraction upon interference with PGE (Fig 3C). These results suggest that both DXS and DXR enzymes might accumulate in an enzymatically active form when PGE is disrupted. To confirm this conclusion, we first quantified the resistance of *rif10-2* and *svr8-2* mutants to FSM. When present in the growth medium, this MEP pathway inhibitor causes concentration-dependent developmental arrest and chlorophyll loss (Fig 1B). Both phenotypes are alleviated when DXS or DXR activity are artificially increased in transgenic plants [21,26,32]. FSM resistance had also been shown for the *rif10-2* mutant [25]. Quantification of both seedling establishment and chlorophyll levels in plants grown with 30 μM FSM confirmed that resistance to the inhibitor was increased in *rif10-2* but also *svr8-2* compared to the WT (Fig 4), consistent with the presence of higher levels of active DXS and DXR enzymes in the mutants (Fig 3). Improved seedling establishment and higher chlorophyll levels were also observed in WT plants when the growth medium containing 30 μM FSM was additionally supplemented with 15 μM LIN to disrupt PGE (Fig 5). These results are consistent with previous observations that FSM resistance of several plant species (including Arabidopsis) can be improved by supplementing the growth medium with sublethal concentrations of the PGE inhibitor CAP but not with the carotenoid biosynthesis inhibitor NFZ, indicating a PGE-specific effect [25].

The FSM resistance phenotype linked to enhanced accumulation of active (i.e. soluble) DXS and DXR enzymes is virtually identical to that previously observed in mutants impaired in Clp protease activity such as *clpr1-2*, defective in the nuclear-encoded ClpR1 subunit of the catalytic core of the protease [16,21,26] (Fig 4). However, LIN treatment did not affect FSM resistance in *clpr1-2* seedlings (Fig 5A), suggesting that both LIN and Clp protease act in the same pathway eventually upregulating the levels of DXS and DXR enzymes. Together, the data support the conclusion that interference with PGE can cause defects in Clp protease activity (likely via the alteration in the levels of plastome-encoded ClpP1 subunit) that eventually lead to FSM resistance by triggering the accumulation of soluble and enzymatically functional MEP pathway enzymes (DXS and DXR). At least in the case of DXS, this regulatory mechanism involves an enhanced activity of the ClpB3 chaperone to favor the refolding of misfolded and aggregated forms of the enzyme that might otherwise accumulate when their degradation is impaired [16]. Higher ClpB3 levels are expected to also alleviate folding stress of many other protein substrates, therefore impacting other metabolic pathways. In fact, mutants defective in PGE or Clp protease activity (including *clpr1-2*) were identified in a screening for mutants able to green in the presence of the carotenoid pathway inhibitor NFZ [33]. We confirmed that NFZ resistance was also gained by partial disruption of PGE in *rif10-2* and *svr8-2* mutants (Fig 4) as well as in LIN-treated plants (Fig 5B).

### Blockage of PGE triggers accumulation of aggregated proteins in the chloroplast

**Fig 6:**
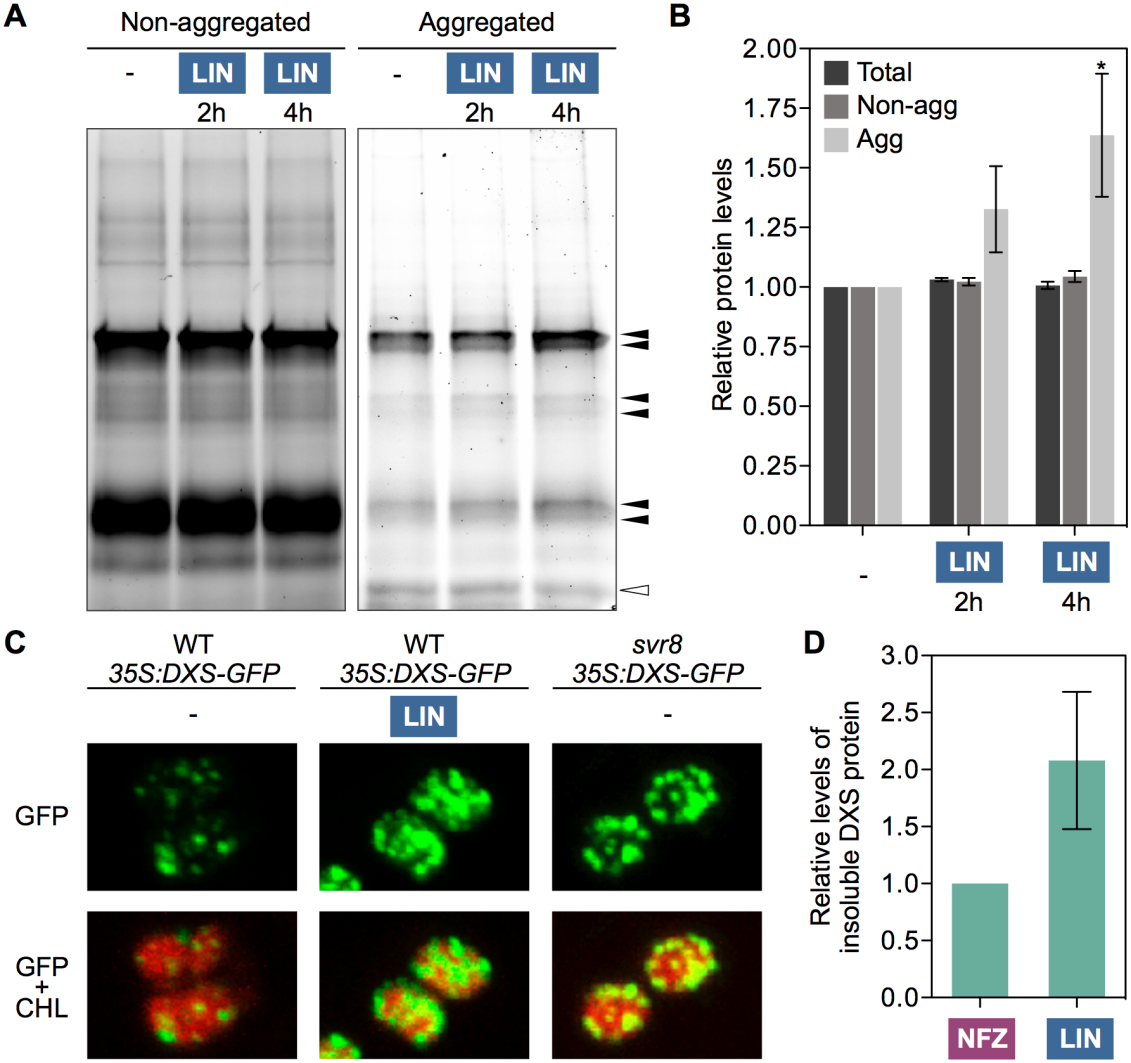
Blockage of PGE results in protein aggregation in chloroplasts. (A) TGX Stain-Free gel showing protein fractions from isolated Arabidopsis chloroplasts treated with LIN for the indicated times. An untreated control is also shown. Following ultracentrifugation of chloroplast lysates, supernatant and pellet fractions were collected (corresponding to non-aggregated and aggregated proteins, respectively) and separated by SDS-PAGE. Bands of aggregated proteins whose intensity increased after LIN treatment are marked with black arrowheads; the white arrowhead marks a major band whose intensity did not increase. (B) Quantification of total protein levels from TGX Stain-Free gel runs corresponding to chloroplast lysates before ultracentrifugation (labeled as “total”) and after separation of non-aggregated and aggregated protein fractions. Protein levels are represented relative to those in untreated controls and correspond to the mean and SEM values of n = 3 independent experiments. Asterisks marks statistically significant difference (*t* test: p<0.05) relative to the untreated sample. (C) Confocal microscopy detection of GFP (green) and chlorophyll (red) fluorescence in chloroplasts of siblings harboring the same T-DNA insertion with the *35S*:*DXS-GFP* construct in WT or *svr8-2* mutant backgrounds. The images were obtained with the same confocal parameters and are to the same scale. They correspond to chloroplasts from the cotyledons of 10-day-old seedlings grown in the presence of absence of 15 μM LIN. (D) Quantification of DXS protein levels detected by immunoblot analysis of insoluble protein fractions isolated from leaves infiltrated with the indicated inhibitors. Results correspond to the mean and SEM values of n = 3 independent experiments are represented relative to those in NFZ-treated samples.

The described results suggested that alteration of chloroplast protein homeostasis following defects in PGE promoted the accumulation of chaperones such as ClpB3, presumably to deal with protein aggregation and folding stress. To confirm whether interference with PGE actually resulted in increased protein aggregation, we analyzed the accumulation of aggregated proteins after treating isolated chloroplasts with 1 mM LIN for several hours to completely inhibit PGE. The rationale of using isolated chloroplasts instead of whole plants was that protein folding stress might be easier to detect in chloroplasts unable to communicate with the nucleus and to increase their folding capacity by importing newly synthesized ClpB3 or other plastid-targeted chaperones. After treatment, chloroplasts were lysed using Triton X-100, a mild non-ionic detergent that solubilizes membrane proteins without denaturing them. Lysates of LIN-treated and untreated (control) chloroplasts were then ultracentrifuged to separate soluble and membrane proteins (supernatant) from insoluble aggregates (pellet). As predicted, blockage of PGE after addition of LIN to the chloroplast preparation led to increased accumulation of aggregated proteins only hours after treatment (Fig 6A and 6B).

In the case of DXS, the amount of protein in insoluble fractions was higher in LIN-grown and PGE-defective seedlings compared to untreated WT controls (Fig 3C). Enhanced association of DXS proteins with insoluble fractions was also observed in mutants impaired in Clp protease [16,21]. Consistent with the conclusion that this likely results from enhanced protein aggregation, the characteristic DXS-GFP aggregates observed as plastidial fluorescent spots in *35S*:*DXS-GFP* lines [19] increased when PGE was impaired in LIN-grown or *svr8-2 35S*:*DXS-GFP* seedlings (Fig 6C). Immunoblot analyses of DXS accumulation in insoluble fractions from leaves infiltrated for 3h with either 400 μM LIN or 400 nM NFZ (as a PGE-unrelated control) confirmed that DXS aggregates were formed *in planta* soon after inhibiting PGE (Fig 6D).

### Treatment with LIN elicits a rapid upregulation of nuclear genes encoding plastid-targeted chaperones

If the response to PGE defects is part of a true cpUPR mechanism, it would be expected that the protein aggregation stress caused by LIN treatment would rapidly trigger changes in the expression of nuclear genes encoding the corresponding plastidial chaperones. To test this possibility, we analyzed the expression of the Arabidopsis genes encoding ClpB3 and Hsp70 chaperones, as they can act synergistically to prevent the wasteful accumulation of insoluble aggregates [22,23,34]. We also analyzed the expression of the nuclear gene encoding Hsp21 (also known as HSP25.3-P), a plastid-localized small heat shock protein proposed to provide fast protection against stress-induced protein aggregation in cooperation with Hsp100 and Hsp70 chaperones [35–37]. As shown in S2 Fig, genes encoding Hsp21, ClpB3, and the two plastidial Hsp70 isoforms found in Arabidopsis, Hsp70.1 and Hsp70.2 [38,39], are rapidly but transiently induced after a heat shock, when protein folding stress occurs in all cell compartments. After a brief lag period (30 min) in which gene expression does not change, transcripts encoding ClpB3 and Hsp70.2 peak at 1h, earlier than those for Hsp70.1 (3h). They all return to normal levels relatively soon (6h) after heat treatment (S2A Fig). By contrast, *Hsp21* transcript levels already increase 2-fold at 30 min, reach about 3,000-fold higher levels at 3h, and still remain higher than before the treatment (40-fold) after 12h (S2B Fig). Genes encoding Clp protease subunits of the catalytic core (S2C Fig) or the chaperone domain of the complex, including ClpC1 (S2D Fig), remained unchanged. *DXS* gene expression was also unaffected by the heat treatment (S2D Fig).

**Fig 7:**
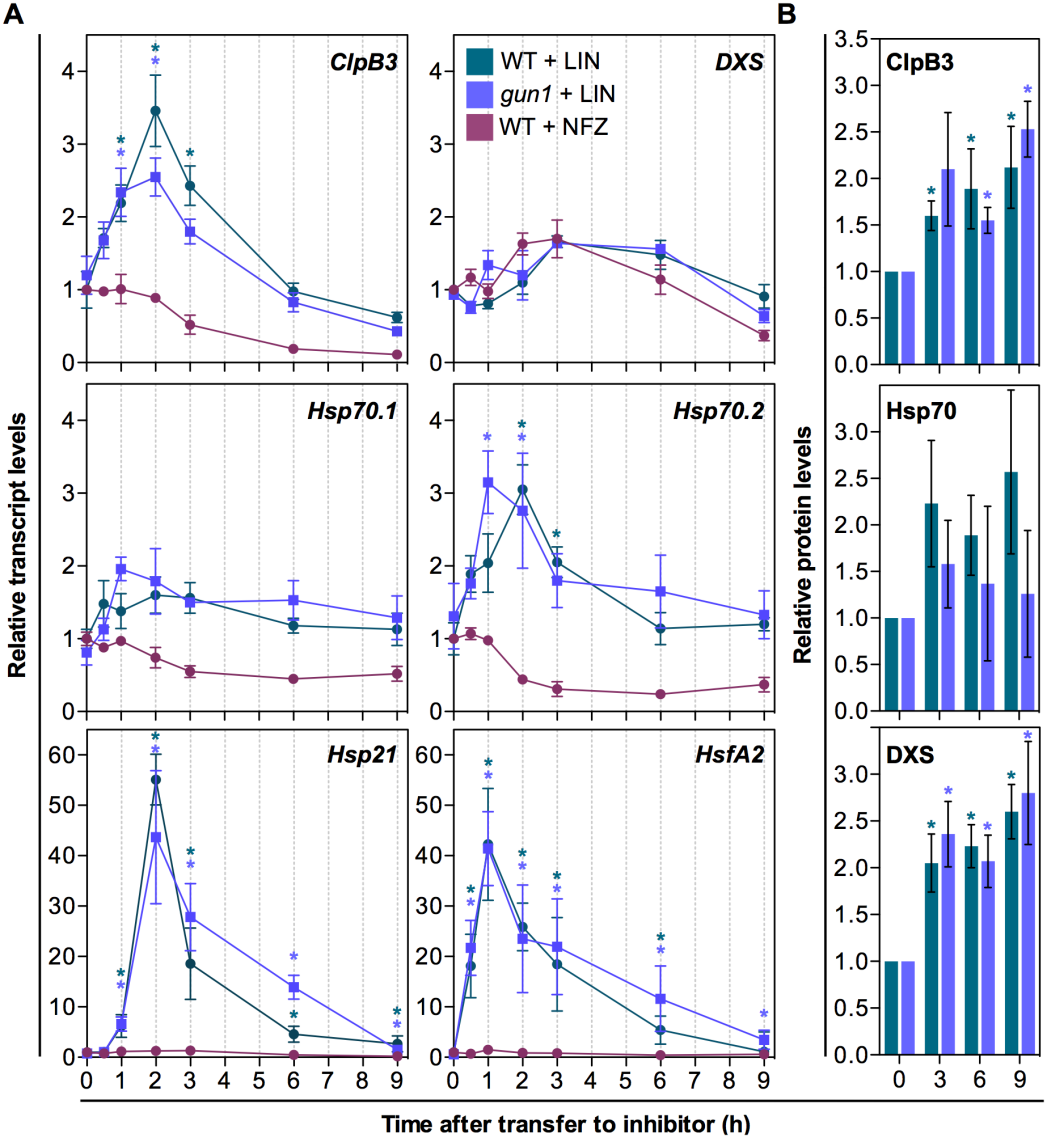
Interference with PGE triggers a rapid but transient expression of specific genes. (A) Quantitative RT-PCR (qPCR) analysis of transcript levels of the indicated genes in 7-day-old WT and *gun1-101* plants after transferring to medium with 400 μM LIN or 400 nM NFZ. (B) Levels of ClpB3, Hsp70 and DXS proteins detected by immunoblot analysis in LIN-treated samples. Data correspond to the mean and SEM values of n≥3 independent experiments, and asterisks mark statistically significant differences (*t* test: p<0.05) relative to untreated (0h) samples.

**Fig 8:**
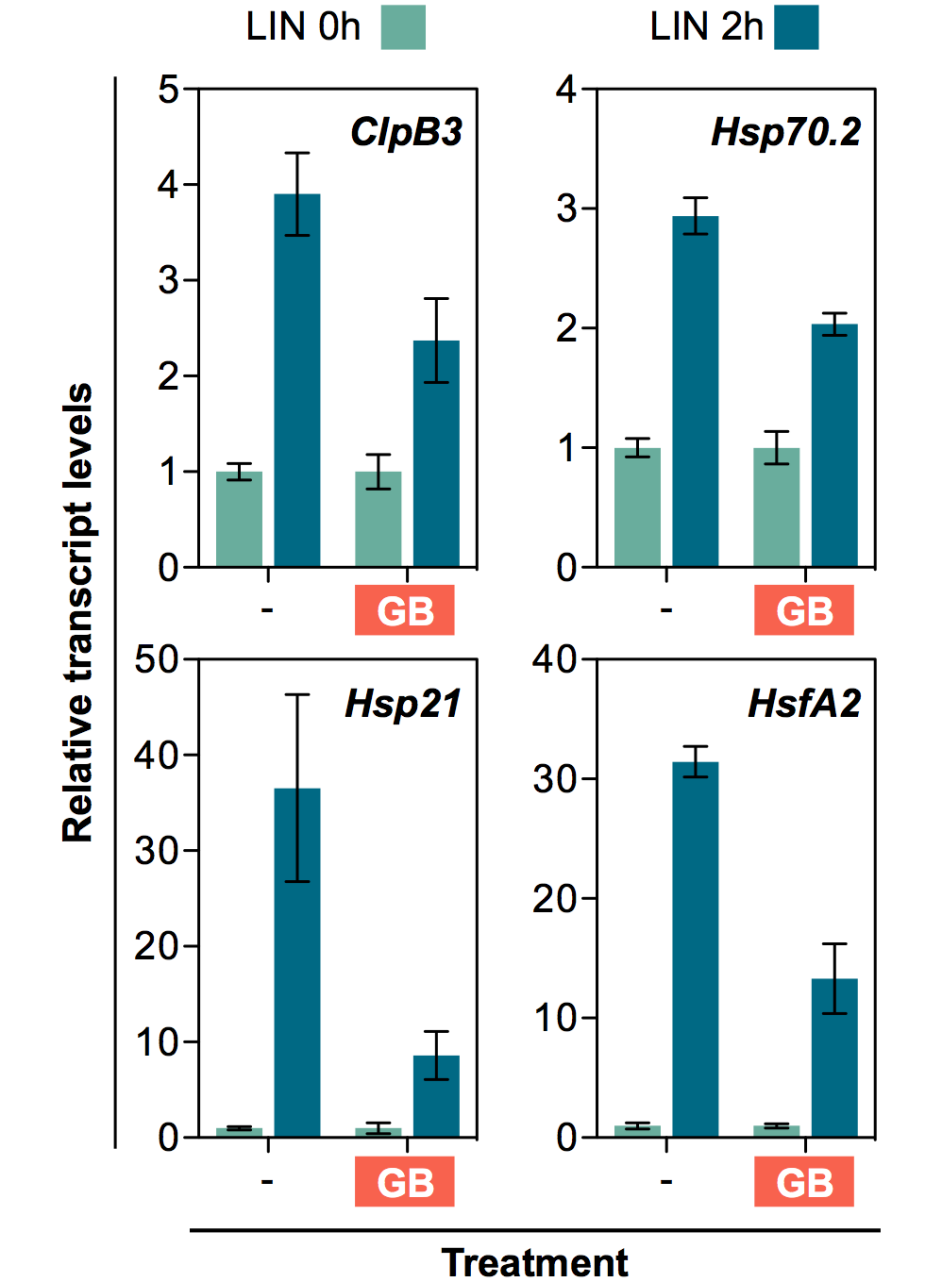
Pre-treatment with a chemical chaperone desensitizes the molecular response to LIN. Quantitative (qPCR) analysis of transcript levels of the indicated genes in 7-day-old WT plants grown in the presence or absence of glycine betaine (GB) and then transferred for 2h to fresh medium supplemented with 400 μM LIN. Data correspond to the mean and SEM values of n = 3 independent experiments and they are represented relative to the levels before the LIN treatment. For all the genes tested, statistically significant differences (*t* test: p<0.05) were found between the LIN-triggered induction of control and GB-grown samples.

To test whether plastidial protein folding stress caused by LIN also had the capacity to elicit changes in the expression of specific nuclear genes (e.g. those encoding plastid-targeted chaperones that alleviate this stress), we designed an experiment to trigger the response at a certain timepoint and then follow the accumulation of selected transcripts and proteins. We grew WT plants on a mesh placed on top of solid growth medium for 7 days under LD conditions. Then, we transferred the mesh with the seedlings to fresh medium supplemented with 400 μM LIN to ensure a rapid inhibition of PGE. To distinguish between PGE-specific and unspecific effects, we did a similar experiment using 400 nM NFZ instead of LIN. Samples were collected at different time points after transfer to extract RNA and protein for quantitative real-time PCR and immunoblot analysis, respectively (Fig 7). A transient accumulation of transcripts encoding ClpB3 and Hsp70.2 was detected after LIN treatment, but not in NFZ-exposed seedlings (Fig 7A). Transcript levels peaked at 2h and then returned to initial levels by 6h. *Hsp21* transcripts were also upregulated by LIN but not by NFZ, confirming that the observed effect is specifically caused by direct interference with PGE. In the case of *Hsp21*, however, the induction was much more dramatic (about 50-fold at the 2h peak, compared to 3-fold in the case of *ClpB3* and *Hsp70*.*2*) and they remained higher (3-fold) after 9h (Fig 7A), somehow paralleling that observed in the response to heat shock (S2 Fig). By contrast, transcripts for Hsp70.1 only showed minor changes after exposure to LIN (Fig 7A). Most interestingly, seedlings germinated and grown in the presence of glycine betaine, a plastid-synthesized chemical chaperone that protects proteins against stress [40,41], showed a decreased up-regulation of *ClpB3*, *Hsp70*.*2* and *Hsp21* expression after exposure to LIN (Fig 8), suggesting that alleviation of protein aggregation desensitizes the retrograde pathway involved in this response.

At the protein level, both ClpB3 and Hsp70 chaperones tended to progressively accumulate in LIN-treated samples (Fig 7B). However, statistical analysis only detected significant (*p*<0.05) differences between untreated (0h) and LIN-treated samples in the case of ClpB3. Higher levels of DXS protein were also found in LIN-treated plants (Fig 7B). In agreement with the conclusion that the increase in DXS protein levels is a consequence of interfering with PGE and eventually down-regulating Clp protease activity, DXS-encoding transcripts were found to oscillate during the timeframe of the experiment (as expected based on the reported circadian control of *DXS* gene expression; [42]) but not to differentially respond to LIN (Fig 7A). Similarly, the level of transcripts for the Clp proteolytic core protein ClpR1 and the ClpC1 chaperone remained virtually unchanged in response to LIN (S3 Fig). This result, together with the lack of response of these and other genes encoding Clp protease subunits to heat shock (S2 Fig), suggests that episodes of protein misfolding and aggregation in chloroplasts do not elicit changes in this proteolytic complex. Based on the described results, we conclude that LIN-mediated interference with PGE results in protein aggregation and elicits a retrograde transcriptional response aimed to deal with protein folding stress, the characteristic features of a cpUPR. This response might eventually allow the proteins that fail to be degraded by a saturated Clp protease to remain correctly folded (e.g. soluble in the case of DXS) and hence active.

### A GUN1-independent retrograde pathway controls cpUPR-related gene expression via the heat shock transcription factor HsfA2

**Fig 9:**
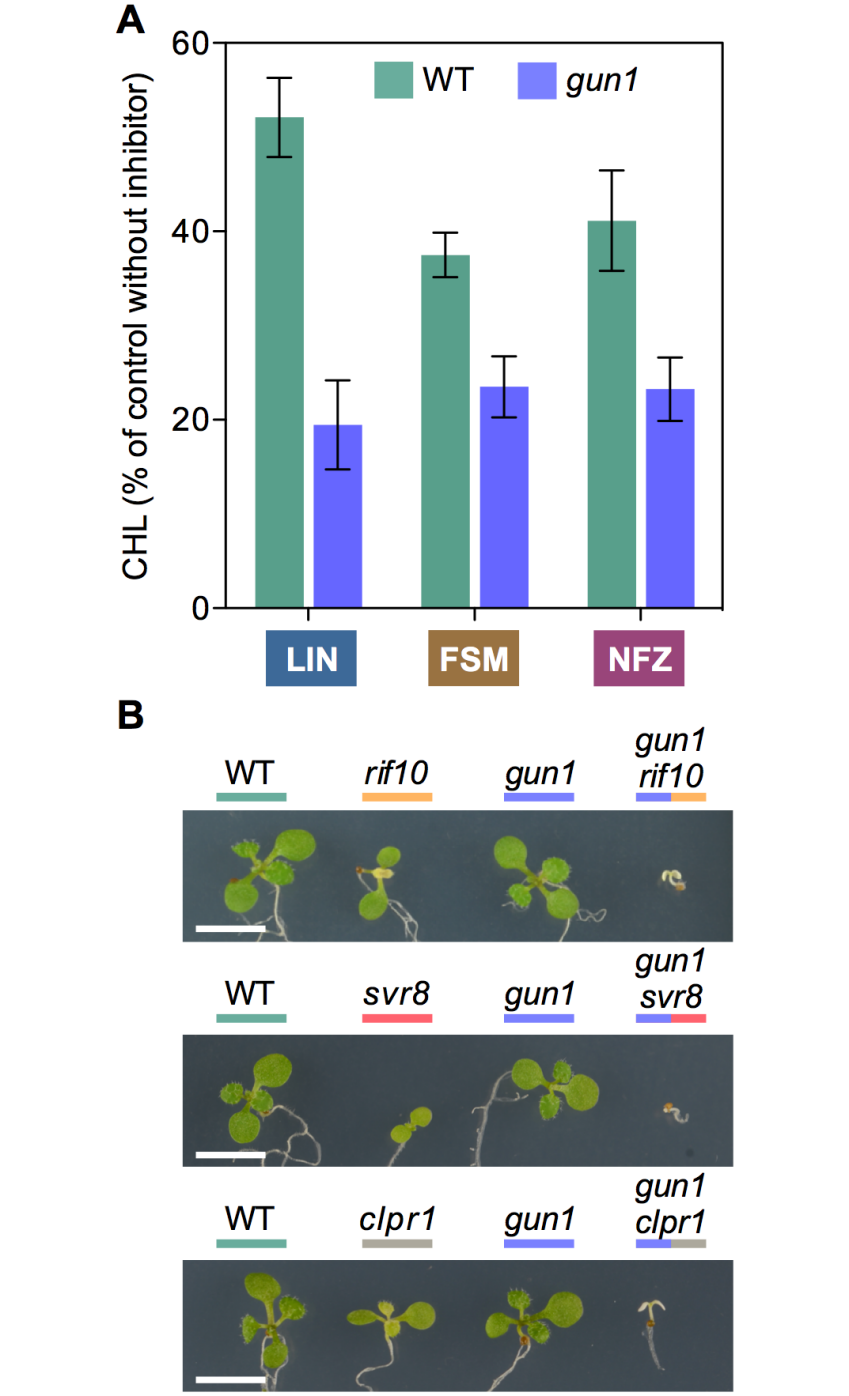
GUN1 contributes to chloroplast protein homeostasis and is required for survival of PGE-defective mutants. (A) Resistance of WT and *gun1-101* plants to the indicated inhibitors estimated as CHL level of plants germinated and grown in the presence of 15 μM LIN, 30 μM FSM or 35 nM NFZ relative to no-inhibitor controls. Data correspond to the mean and SEM values of n≥3 independent experiments. (B) Phenotype of 10-day-old WT and mutant lines of the indicated genotypes grown in the same plate. Bar, 5 mm.

Several retrograde signals and pathways have been reported in the literature to regulate nuclear gene expression when chloroplast normal functions are compromised [2–4]. The plastidial pentatricopeptide repeat protein GUN1 integrates multiple retrograde signals (including those related to PGE) and has been recently proposed to participate in a putative cpUPR signaling pathway [43]. However, GUN1-defective plants of the knock-out *gun1-101* allele [44] showed a virtually WT profile of chaperone gene expression after LIN treatment (Fig 7A). These results suggest that GUN1 is not required to produce or/and transduce the PGE-related plastidial signal that eventually regulates nuclear gene expression in LIN-treated seedlings. Strikingly, the *gun1-101* mutant was unable to respond to LIN treatment in terms of improving FSM resistance (Fig 5A) despite having a WT phenotype of LIN-induced accumulation of ClpB3 and DXS proteins (Fig 7B). GUN1 was recently found to interact with proteins rather than nucleic acids [45]. GUN1 interactors include proteins related to PGE (such as components of the plastome transcription, RNA editing, and translation machinery) and PQC (including the Hsp70 and ClpC chaperones present in the chloroplast). It is therefore conceivable that GUN1 acts as a coordinator of PGE, PQC, and cpUPR at the protein-protein interaction level to ensure that protein homeostasis is properly maintained in chloroplasts exposed to environmental challenges [43,45]. Consistent with this possibility, the *gun1-101* mutant shows increased sensitivity to a partial blockage of PGE with LIN, the MEP pathway with FSM, or the carotenoid pathway with NFZ, compared to WT seedlings (Fig 9A). A genetic confirmation of this central role of GUN1 came from the analysis of double mutants with *gun1-101* and mutants defective in PGE and PQC (*clpr1-2*, *rif10-2* and *svr8-2*). In all the cases, double mutants germinated but were unable to develop beyond the cotyledon stage (Fig 9B).

Based on the expression profile of chaperone-encoding genes, the response to PGE disruption with LIN is similar (but weaker) to that observed after a heat shock, perhaps because both involve protein aggregation but the latter affects all cell compartments. The response to heat stress is orchestrated at the transcriptional level by heat shock transcription factors such as HsfA2. Because Arabidopsis HsfA2 has been shown to participate in heat-responsive retrograde pathways [46] and to directly induce genes encoding plastidial chaperones such ClpB3 and Hsp21 [47,48], we investigated the participation of this transcription factor in the cpUPR mechanism. The level of *HsfA2* transcripts dramatically increased after exposing WT seedlings to LIN (but not in response to NFZ) and, consistent with a role for HsfA2 in up-regulating *ClpB3* and *Hsp21* expression, *HsfA2* induction preceded that of the chaperone-encoding genes (Fig 7A). Again, this is a similar but weaker response compared to heat shock (S2 Fig). As expected based on the behavior of target genes *ClpB3* and *Hsp21*, the induction of *HsfA2* gene expression in response to LIN was repressed by glycine betaine (Fig 8) and did not require the activity of GUN1 (Fig 7A).

## Discussion

**Fig 10:**
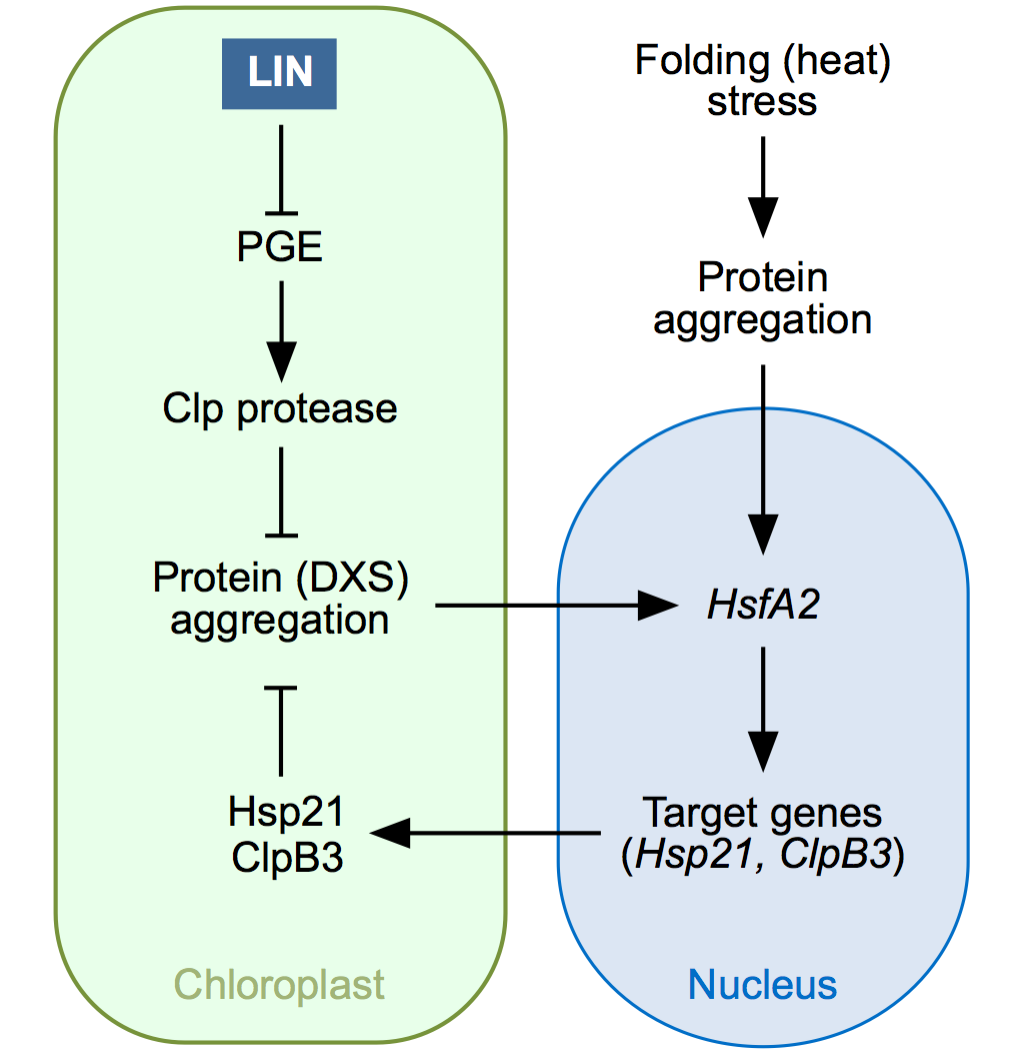
Model for the cpUPR mechanism in Arabidopsis. LIN treatment represses PGE and this eventually causes a reduced activity of the Clp protease. Normal Clp protease activity removes misfolded proteins and hence prevents protein aggregation. When Clp protease activity is compromised, however, the misfolded proteins that fail to be degraded (including DXS) aggregate. Build-up of protein aggregates somehow sends an unknown retrograde signal to upregulate the expression of *HsfA2*, a gene that can also be induced (at much higher levels) by heat stress episodes causing protein aggregation in other cell compartments. *HsfA2* encodes a transcription factor that in turn induces the expression of target genes encoding chloroplast chaperones such as *Hsp21* and *ClpB3*. As a result, more of these chaperones are synthesized and imported into plastids, eventually contributing to alleviate protein folding stress in this organelle.

In this study, we provide new evidence strongly supporting the existence of a cpUPR in Arabidopsis. Based on our results and published information from other systems, we propose the model presented in Fig 10. In brief, LIN treatments and stress conditions that disrupt PGE or/and cause protein aggregation (e.g. by overwhelming Clp protease activity) unleash a specific retrograde signaling pathway to rescue proteostasis by up-regulating *HsfA2* and downstream nuclear target genes such as *Hsp21* and *ClpB3*. As a consequence, folding capacity is increased to restore functional integrity of chloroplast proteins such as DXS, the main rate-determining enzyme of the MEP pathway. We also demonstrate that GUN1 does not participate in the regulation of this transcriptional response but most likely acts as an integrator of the chloroplast PQC machinery.

### A dynamic mechanism to balance protein degradation and repair

The observed cpUPR was triggered by using the PGE inhibitor LIN. The first link between altered PGE, reduced Clp protease activity, and enhanced accumulation of MEP pathway enzymes (including DXS) was provided by the isolation of FSM-resistant (*rif*) mutants such as *rif1* [26,27] and *rif10* [25]. Here we report that a *rif* phenotype is also observed in other PGE mutants such as *svr8-2* (Figs 3 and 4) and can be induced in WT plants by partially inhibiting PGE with sublethal concentrations of LIN (Figs 3 and 5), similar to that previously described using CAP [25]. The observation that FSM resistance of *clpr1-2* seedlings does not change in the presence of concentrations of LIN that do improve FSM resistance in the WT (Fig 5A) further supports the conclusion that PGE defects and the Clp protease act in the same pathway eventually resulting in the accumulation of active MEP pathway enzymes. When the capacity of the Clp protease to remove non-functional proteins is compromised (e.g. by treatment with LIN or in mutants), the excess accumulation of aggregated proteins appears to trigger a cpUPR mechanism to rescue protein homeostasis (Fig 10). We show that chloroplast proteostasis is carried out by a dynamic balance between degradation and repair of structurally compromised proteins. In the case of DXS, degradation is specifically carried out by the Clp protease with the involvement of Hsp70 and ClpC1 unfolding chaperones, whereas the combined unfolding and disaggregating actions of Hsp70 and ClpB3 chaperones allow solubilization and hence reactivation of the enzyme [16,19] (Fig 1A). The characteristic M domain of ClpB3 allows direct interaction with Hsp70 [16]. Unlike that observed in bacterial homologues (such as *E*. *coli* ClpA), this domain is not completely absent in Arabidopsis ClpC1 [16], raising the possibility that the unfolding of substrates for degradation by the plastidial Clp protease complex might also be under the direct control of Hsp70. In agreement, Hsp70 and ClpC1 chaperones have been co-immunoprecipitated, suggesting a direct (or close) interaction [49,50].

It was proposed that the relative abundance of ClpC1 and ClpB3 might determine the eventual fate of DXS and other Hsp70-delivered client proteins [16]. Under normal growth conditions, the levels of ClpB3 transcripts and proteins are much lower than those of ClpC1 and hence it would be expected that damaged or inactivated (misfolded) forms of DXS were preferentially degraded (Fig 1A). Here we show that when the degradation capacity of this system is compromised, a cpUPR diverts the Hsp70-mediated unfolding of DXS towards the recovery pathway by upregulating ClpB3 (but not ClpC1). The existence of these opposite pathways has major biological implications. Degrading misfolded and aggregated chloroplast proteins such as DXS not only consumes energy for Hsp70 and ClpC1-mediated unfolding and subsequent Clp protease degradation but it also involves a high cost to resynthetize new proteins, import them into the chloroplast, and properly fold them to substitute the ones that were removed. In contrast, disaggregation and refolding of DXS proteins that lose their active conformation in the chloroplast by the alternative Hsp70-ClpB3 pathway ensures that the protein function will be restored in a much faster and energy-saving way. While this adaptive mechanism might be particularly useful under stress conditions, its fundamental relevance for normal plant development is illustrated by the seedling lethal phenotype of double mutants impaired in both Clp protease (i.e. degradation) and ClpB3 (i.e. reactivation) activities [13].

### Reciprocal regulation of PGE and protein degradation in chloroplasts

The Clp protease not only regulates the accumulation of MEP pathway enzymes but it impacts many other processes in chloroplasts [51]. In particular, reduced Clp proteolytic activity in mutants causes accumulation of many plastidial proteins specifically involved in PGE, including components of the RNA processing and editing as well as protein translation machinery [51]. Therefore, it is tempting to speculate that the two-way reciprocal regulation of PGE and Clp protease might be a compensatory mechanism. Thus, defects in PGE would result in decreased Clp protease activity, which in turn would lead to increased levels of PGE-related proteins to regain overall protein homeostasis in individual plastids (i.e. without the need to regulate nuclear gene expression). In agreement with this model, concentrations of LIN that hardly produced visual symptoms were able to dramatically increase the levels of DXS (Fig 2), suggesting a relatively large decrease in Clp protease activity (i.e. in the capacity to degrade DXS) in response to moderate alterations of PGE. Higher concentrations of LIN led to a concomitant increase in the levels of plastidial chaperones such as ClpB3 (Fig 2), likely because failure to achieve protein homeostasis elicits a cpUPR mechanism to release protein folding stress with the help of nuclear-encoded chaperones.

### cpUPR and increased protection against protein folding stress

Similar to the Arabidopsis cpUPR described here, reduction of Clp protease activity by controlled depletion of ClpP1 in *Chlamydomonas reinhardtii* [8] caused up-regulation of both transcript and protein levels for small heat shock proteins (including several Hsp21 homologues) and chaperones such as Hsp70B and ClpB3, the only plastidial homologues of the Hsp70 [52] and ClpB-type Hsp100 [22] families in the alga. We detected transiently increased accumulation of transcripts encoding Arabidopsis Hsp21, ClpB3 and Hsp70.2 (but not those encoding Hsp70.1) when WT plants were exposed to LIN (Fig 7A). However, our immunoblot analysis was not able to detect Hsp21 proteins and only detected a clear increase in protein levels in the case of ClpB3 (Fig 7B). While Hsp70 chaperones also appeared to accumulate at higher levels after LIN treatment (Fig 7B), statistical analysis did not allow to conclude that such differences were significant, mostly due to large differences between replicates. The commercial anti-Hsp70 serum that we use, raised against both Arabidopsis Hsp70.1 and Hsp70.2 isoforms (http://www.agrisera.com/en/artiklar/hsp70-heat-shock-protein-70-chloroplastic-.html), is presumed to be specific for plastidial Hsp70 proteins. This antibody, however, failed to detect higher Hsp70 levels in Clp protease mutants such as those defective in ClpR1 or ClpC1 [16], whereas proteomic approaches have consistently detected increased levels of these chaperones in *clpr1* [12], *clpc1* [17], and other Clp-defective Arabidopsis mutants [51]. We therefore conclude that our immunoblot analysis might not be sensitive enough to detect actual changes in plastidial Hsp70 levels. In any case, it is remarkable that the putative cpUPR-mediated elevation of Hsp70 chaperone supply to the chloroplasts of LIN-treated WT plants might rely mostly on the up-regulation of the *Hsp70*.*2* gene (Fig 7A). Genes encoding Hsp70.1 and Hsp70.2 are expressed at similar levels in photosynthetic tissues under normal growth conditions [38]. But in response to heat stress, the *Hsp70*.*2* gene is activated faster than *Hsp70*.*1* [39] (S2 Fig). Arabidopsis mutants defective in Hsp70.2 do not show a visual phenotype, but those impaired in Hsp70.1 show variegation and delayed growth despite they accumulate Hsp70.2 proteins at levels higher than those of the two Hsp70 chaperones combined in the WT [38]. It is therefore possible that Hsp70.1 preferentially plays housekeeping functions while Hsp70.2 might be more specialized in responding to plastidial protein folding stress.

Unlike LIN, NFZ treatment did not trigger the accumulation of chaperone transcripts (Fig 7A). While both inhibitors cause similar bleaching symptoms (Fig 1), only LIN has a direct impact on PGE [28–30]. Blockage of carotenoid biosynthesis with NFZ can eventually alter PGE as it leads to decreased photoprotection and photooxidation, but LIN directly inhibits the translation of plastome-encoded proteins and has a much stronger impact on RNA transcription and processing than NFZ [30,53]. In any case, the secondary effects of NFZ on PGE are not expected to be relevant in green plants at short times like those used to analyze the existence of a cpUPR after transferring WT plants to inhibitor-supplemented media (Fig 7A). The absence of a cpUPR in NFZ-treated plants was also deduced from the lack of a *rif* phenotype of enhanced FSM resistance when NFZ was added to the growth medium of WT plants [25]. These results further support the contribution of PGE-triggered changes in Clp protease activity to the cpUPR (Fig 10). NFZ has been widely used to identify retrograde signals and pathways communicating the chloroplasts with the nucleus [1–4]. For example, *genomes uncoupled* (*gun*) mutants, including *gun1* [54], were identified based on their ability to de-repress the expression of nuclear genes encoding photosynthetic proteins in NFZ-supplemented medium. These studies were typically conducted using very high (μM) concentrations of the inhibitor that caused massive photooxidative damage and complete bleaching. By contrast, screening for *happy on norflurazon* (*hon*) mutants able to green in the presence of lower (nM) concentrations of NFZ showed that mutants with altered PGE (*hon23*) or Clp protease activity (such as *hon5*, defective in the ClpR4 subunit of the complex) gained resistance to NFZ [33], consistent with our results using *rif10-2*, *svr8-2*, and *clpr1-2* mutants (Fig 4). It was concluded that perturbance of chloroplast protein homeostasis in *hon* mutants caused a relatively mild stress that led to an activated protection against further stress such as that imposed by NFZ treatment [33]. This proposed stress acclimatization response might be related with the cpUPR unveiled here.

Mutants defective in PGE and Clp protease activity were also repeatedly identified in screenings for Arabidopsis mutants with a phenotype of *suppressor of variegation* (*svr*) of the *yellow variegated 2* (*var2*) mutant, defective in one of the subunits of the chloroplast FtsH protease complex [55,56]. In particular, *svr1* [57], *svr3* [58], *svr4* [59], *svr7* [60], *svr8* [31], *svr9* [61], and *svr10/rif1* [62] are defective in PGE processes such as RNA editing or protein translation, whereas those impaired in Clp protease activity include *svr2/clpr1* and *clpc1* [57]. Furthermore, PGE inhibitors such as CAP [57] and LIN (S4 Fig) can also suppress *var2* variegation. The existence of a cpUPR in Arabidopsis as supported here could explain why interference with PGE and Clp protease activity generates *rif*, *hon* and *svr* phenotypes, as they can be considered as ultimate consequences of triggering a PQC-based stress protection mechanism in chloroplasts. Thus, higher levels of plastidial chaperones such as ClpB3 in *rif10-2*, *svr8-2*, or *cpr1-2* [16] (Fig 3) would contribute to remove protein aggregates and to maintain chloroplast proteins in a correctly folded form, hence preventing their degradation and eventually resulting in enhanced resistance to herbicides such as FSM or NFZ (Fig 4). Increased chaperone levels might also mitigate the deleterious effects produced by accumulation of VAR2 substrates as misfolded polypeptides and protein aggregates, hence causing a reversion of the variegation phenotype of the *var2* mutant.

### HsfA2 might integrate signals from heat shock and cpUPR signaling pathways

Besides confirming the existence of a cpUPR in plants, our work has unveiled some of the molecular components of the signal transduction pathway (Fig 10). In particular, the kinetics of transcript accumulation following sudden inhibition of PGE with LIN (Fig 7A) suggests that the chloroplast signal first regulates the expression of the nuclear gene encoding the transcription factor HsfA2 (peaking 1h after LIN treatment). Then, HsfA2 induces the expression of target genes, including those encoding Hsp21 and ClpB3 (with a peak at 2h). It is possible that *Hsp70*.*2* might also be a gene regulated by HsfA2 (either directly or indirectly), as it also peaks at 2h (Fig 7A). Strikingly, the gene expression response to LIN is very similar (but weaker) to that observed after a heat shock (S2 Fig). As heat stress causes protein aggregation in all cell compartments, including the chloroplast, it is possible that the enhanced response to heat shock in terms of *HsfA2* and chaperone gene expression was the consequence of converging signaling pathways triggered by protein aggregation in different cell locations (Fig 10). This model predicts that heat stress should also increase the refolding capacity of chloroplasts, as it induces the expression of *HsfA2* and downstream genes encoding plastid-targeted chaperones. In agreement, exposure of WT seedlings to heat dramatically improved their resistance to FSM (S5 Fig). The observation that *Chlamydomonas* lines with reduced levels of the HsfA2 homologue HSF1 failed to induce the expression of genes encoding plastid-targeted chaperones after a heat shock [63] further suggests that these transcription factors are conserved targets of the cpUPR-associated retrograde signal.

### Mechanistics insights into the retrograde pathways involved in cpUPR

Several retrograde signaling pathways have been proposed to mediate the communication of chloroplast stress (including heat stress) to the nucleus [1–4]. GUN5, a protein involved in tetrapyrrole-mediated signaling, was recently shown to mediate the retrograde control of *Hsp21* gene expression under heat stress [64]. Interestingly, heat-triggered induction of *HsfA2* expression is also regulated by retrograde signals [46]. Because a GUN5-dependent tetrapyrrole metabolite has been shown to inhibit the activity of cytosolic Hsp90 chaperones [65] and Hsp90 binds *Chlamydomonas* HSF1 to presumably prevent its activity [9], it is tempting to speculate that a tetrapyrrole produced by stressed chloroplasts might decrease Hsp90 activity, hence releasing HsfA2 to regulate cpUPR-related gene expression. However, *gun5* mutants were found to normally induce *ClpB3* expression in response to LIN (S6 Fig). Besides the tetrapyrrole-dependent pathway, GUN1 integrates signals derived from perturbations in two other major retrograde pathways: PGE and redox homeostasis [1–4]. The observation that *gun1-101* plants showed a WT profile of LIN-mediated gene expression and protein accumulation in response to LIN treatment (Fig 7), however, suggests that this central integrator of retrograde pathways is not required to trigger cpUPR-related transcriptional responses. In agreement with this conclusion, mutants defective in ABI4, a transcription factor involved in GUN1-dependent chloroplast retrograde signaling [54], also showed a normal induction of *ClpB3* expression in response to LIN (S6 Fig). However, the inability of *gun1-101* plants to unfold the subsequent acclimatory response (Fig 5A) and to efficiently cope with stress caused by inhibition of chloroplast function [33] (Fig 9A) as well as the seedling lethal phenotype of double mutants defective in GUN1 and PGE or PQC (Fig 9B) strongly suggest that the GUN1 protein is a pivotal component of the overall cpUPR response at the protein level. This is consistent with the observation that GUN1 interacts with many proteins involved in PGE and PQC processes [45] and with the conclusion that GUN1 is a coordinator of chloroplast PGE, protein import, and protein homeostasis [43]. It has been proposed that GUN1 might act as a platform to bring different protein together to promote or/and prevent particular interactions [43].

Among the GUN1-independent retrograde signals, isoprenoid-related metabolites such as the MEP pathway intermediate methylerythritol cyclodiphosphate (MEcPP) and carotenoid-derived products such as β-cyclocitral were found to participate in stress responses [66,67]. In particular, MEcPP (Fig 1A) mediates the rapid and transient induction of general stress response genes and triggers the endoplasmic reticulum (ER) UPR in advance of the accumulation of misfolded proteins in this cell compartment [68,69]. If any of these isoprenoid metabolites were involved in the cpUPR, it would be expected that their differentially altered levels in WT plants treated with FSM or NFZ (Fig 1A) resulted in opposite responses to LIN. However, plants growing with FSM or NFZ showed a similar response to LIN (Fig 5B). Arabidopsis mutants accumulating high levels of MEcPP also showed a WT response to LIN treatment in terms of gene expression (S6 Fig) and WT levels of plastidial chaperones (ClpB3 and Hsp70) and MEP pathway enzymes (DXS and DXR) at both transcript and protein levels (S7 Fig). Transcripts encoding HsfA2 and Hsp21 were also unaltered MEcPP-overaccumulating mutants (S7 Fig). Together, an involvement of MEcPP on the transduction pathway activating cpUPR is unlikely.

While much work is still ahead to unveil the detailed molecular pathway connecting disturbed proteostasis in the chloroplast with increased expression of *HsfA2*, work in *Caenorhabditis elegans* and mammals suggest several possibilities based on mitochondrial and ER UPR mechanisms [6,7,70]. In *C*. *elegans*, the small peptides that result from degradation of protein clients by the mitochondrial Clp protease are exported to prevent mitochondrial import and promote nuclear translocation of ATFS-1, a bZIP transcription factor that orchestrates expression of mitochondrial UPR-related genes [18,71,72]. Plant ER UPR also relies on the differential targeting of bZIP transcription factors, namely bZIP60 and bZIP28. They are normally anchored to the ER under non-stressed conditions. However, accumulation of misfolded proteins in the ER lumen triggers BiP chaperone-dependent pathways eventually producing nuclear-targeted versions of these transcription factors by differential splicing (bZIP60) or proteolytic cleavage (bZIP28) [70]. Some plastidial retrograde signaling pathways also rely on transcription factors that can relocate from the chloroplast to the nucleus. Among them, the homeodomain transcription factor PTM is normally anchored to the chloroplast envelope but upon a GUN1-mediated stimulus translocates to the nucleus, where it enhances *ABI4* expression [73]. The observation that neither GUN1 nor ABI4 are required for normal cpUPR-associated gene expression (Fig 7A and S6 Fig) suggests that this response might also be independent of PTM. WHIRLY1 (WHY1) is another example of transcription factor with alternate plastid-nucleus localization [74]. It has been proposed that changes in the redox state of the chloroplast cause destabilization of WHY1 oligomers and release of monomeric proteins, which are then translocated to the nucleus to regulate transcription [75]. Interestingly, WHY1 is involved in maintaining plastome stability [76] and accumulates in ClpC1-defective mutants [15]. It is conceivable that protein folding stress resulting from reduced plastome function or/and Clp protease activity (e.g. by LIN treatment) might also destabilize WHY1 oligomers, eventually promoting the nuclear targeting of this transcription factor. A high-throughput quantitative proteomic analysis of chloroplasts and nuclei after LIN treatment should contribute to confirm this hypothesis and identify other dual-localized transcription factors potentially involved in the cpUPR-associated retrograde signaling pathway in Arabidopsis. In combination with RNAseq, it should also provide a comprehensive picture of how this cpUPR impacts chloroplast functions to efficiently overcome stress.

## Methods

### Plant material

*Arabidopsis thaliana* WT, transgenic *35S*:*DXS-GFP* [19], and mutant *rif10-2* (SALK_037353), *svr8-2* (SALK_010822), *clrp1-2* (SALK_088407), *gun1-101* (SAIL_33_D01), *var2-8* [77], *abi4-1* [78], *gun5-1* [79], and *csb3/clb4-3* [80] lines were in the Columbia ecotype. For generation of double mutants, single homozygous mutants were crossed and the F_2_ progeny was screened for the characteristic pale phenotype associated to the *clpr1-2*, *rif10-2* or *svr8-2* mutations in homozygosis. Then, pale individuals were PCR-genotyped to identify the T-DNA insertion of the *gun1-101* allele as described [44]. Individuals confirmed to be homozygous for *clpr1-2*, *rif10-2* or *svr8-2* and heterozygous for *gun1-101* were allowed to self-cross. Double mutants, segregated as tiny albino plants in the F_3_ generation, were confirmed by PCR analysis of the T-DNA insertions in both genes. The *35S*:*DXS-GFP* transgene was introgressed into the *svr8-2* mutant background by cross-fertilization.

### Growth conditions and inhibitor treatments

Seeds were surface-sterilized and germinated on solid 0.5 X Murashige and Skoog (MS) medium without sucrose or vitamins, and plates were incubated in a growth chamber at 22°C under LD conditions as described [19]. For long-term inhibitor treatments, the medium was supplemented with the indicated concentrations of FSM (Life Technologies), NFZ (Zorial) or/and LIN (Sigma). Quantification of resistance was based on seedling establishment (in 14-day-old plants) and chlorophyll levels (in 7 to 10-day-old plants) measured as described [81]. For short-term inhibitor treatments, seeds were germinated on a sterile mesh disc (SefarNitex 03-100/44) placed on top of solid MS medium without inhibitors or supplemented with 10 mM glycine betaine (Sigma). Plates were incubated under LD for 7 days and then the disc with the seedlings was transferred to fresh medium supplemented with 400 μM LIN or 400 nM NFZ. Samples were collected at different time points for qPCR and immunoblot analysis.

Adult WT plants grown on soil for 4 weeks at 22°C under LD conditions were used for inhibitor infiltrations and chloroplast isolation. Rosette leaves were infiltrated with either 400 μM LIN or 400 nM NFZ, and samples were collected 3h later for immunoblot analyses of insoluble fractions. Intact chloroplasts were isolated as described [26]. Equal volumes of the chloroplast suspension were then treated with 1mM LIN or a mock solution of the same volume without the inhibitor and incubated in the light for up to 4h.

### Protein extraction and immunoblot analysis

Total protein extraction, separation of soluble and insoluble fractions from whole seedlings extracts, immunoblot analyses, and quantification of protein abundance were performed as described [16,19]. The following antibodies (and dilutions) were used: DXS (1:500), DXR (1:7,000), Hsp70 (1:7,000), ClpC (1:1,500), and ClpB3 (1: 2,000). The last three antibodies were supplied by Agrisera. For the detection and quantification of protein aggregates, LIN-treated and control chloroplasts were lysed with TKMES buffer [19] supplemented with 0.3% Triton X-100 in a total volume of 3.7 ml. After collecting a 0.2 ml aliquot as the “total” fraction, lysates were subjected to centrifugation at 125,000 x *g* for 30 min. Then, the supernatant (“non-aggregated” fraction) was collected and the pellet (“aggregated” fraction) was directly resuspended in 0.2 ml of SDS-PAGE loading buffer. Equal volumes of each fraction were run on TGX Stain-Free gels (BioRad) to estimate protein content from fluorescence intensity using the Image lab (BioRad) software.

### Gene expression analysis

Total RNA was extracted from whole seedlings using the Maxwell 16 LEV Plant RNA Kit (Promega). RNA was quantified using a NanoDrop (Thermo Scientific) and its integrity was analyzed by agarose gel electrophoresis. cDNA was synthetized using the cDNA Synthesis Kit (Roche). Real-time quantitative PCR was performed in a total reaction volume of 20 μL using LightCycler 480 SYBR Green I Master (Roche) and gene-specific primers (S1 Table) on a LightCycler 480 Real-Time PCR System (Roche). The normalized expression of target genes was calculated using *UBC* as the endogenous reference gene (S1 Table).

### Confocal microscopy

Subcellular localization of DXS-GFP and chlorophyll fluorescence was determined with an Olympus FV 1000 confocal laser-scanning microscope using an argon laser for excitation (at 488 nm) and 500–510 nm filter for detection of GFP fluorescence and 610–700 nm filter for detection of chlorophyll fluorescence. All images were acquired using the same confocal parameters.

## Supporting information

S1 Fig Interference with PGE does not affect *DXS* expression. PGE was partially blocked either pharmacologically (by germinating and growing WT plants in the presence of 15μM LIN) or genetically (*rif10-2* and *svr8-2* mutants). Both WT+LIN and mutant plants showed a similar reduction in chlorophyll levels compared to untreated WT controls (left graph) but no differences in the levels of *DXS* transcripts (right graph). Chlorophyll quantification and *DXS* mRNA levels of 10-day-old WT, WT LIN-treated (15μM), *rif10-2* and *svr8-2*. Data correspond to the mean and SEM values of n = 3 independent experiments.
(TIFF)

S2 Fig Transcript levels of genes encoding stromal chaperones, heat-shock related proteins, Clp protease complex subunits, and DXS after a heat shock. Data were obtained from the Arabidopsis eFP browser at www.bar.utoront.ca and correspond to the gene expression map of Arabidopsis abiotic (heat) stress treatment. Briefly, boxes with Arabidopsis plants grown under LD conditions on polypropylene rafts on MS medium supplemented with 0.5% agar and 0.5% sucrose were transferred from the growth chamber (at 24°C) to an incubator and exposed to a temperature of 38°C for 3h. Then, they were returned to the growth chamber and samples were collected at the indicated times. The results shown correspond to shoot samples.
(TIFF)

S3 Fig Quantitative (qPCR) analysis of the expression of genes encoding ClpR1, ClpC1, ClpB3 and DXS in response to LIN. WT seedlings grown for 7 days on a mesh on top of MS solid medium were transferred to fresh medium containing 400 μM LIN and then whole-plant samples were collected at the indicated timepoints. Transcript levels are represented relative to untreated (0h) samples. Data correspond to the mean and SEM values of n≥3 independent experiments.
(TIFF)

S4 Fig LIN treatment suppresses *var2* variegation. Picture shows representative individuals of WT and *var2* plants germinated and grown for 10 days in the presence or absence of LIN (15μM). Bar, 5 mm.
(TIFF)

S5 Fig Heat stress improves resistance to FSM. (A) Resistance was estimated by quantifying SE of 14-day-old WT seedlings germinated and grown at 22°C on plates of MS media supplemented with 30 μM FSM. Heat treatment was carried out by exposing the plates with the seedlings every day to 37°C for 90min. (B) Representative images of the seedlings.
(TIFF)

S6 Fig Levels of *ClpB3* transcripts in retrograde signaling mutants after LIN treatment. Transcript levels were quantified by qPCR analysis before and after transferring WT and mutant plants to medium with 400 μM LIN for 2h. Levels are represented relative to those in untreated (0h) controls. Data correspond to the mean and SEM values of n = 3 experiments. The *csb3* mutant accumulates abnormally high levels of MEcPP. See Materials and methods for references on the mutants.
(TIFF)

S7 Fig Transcript and protein levels of plastidial chaperones, heat shock related proteins, and MEP pathway enzymes in the MEcPP-accumulating mutant *ceh1*. Levels are represented relative to those in WT plants. Data taken from Walley *et al*. (2015) *Proc Natl Acad Sci USA* 112: 6212–7, Supplemental Dataset S1. Hsp21 and HsfA2 proteins were not detected.
(TIFF)

S1 Table List of primers for qPCR. References are indicated.
(TIF)
