# Supplementary figures and images for: Interference with plastome gene expression and Clp protease activity in Arabidopsis triggers a chloroplast unfolded protein response to restore protein homeostasis
Source: PLoS Genet. 2017 Sep 22;13(9):e1007022. doi: 10.1371/journal.pgen.1007022 (PMC5627961; doi:10.1371/journal.pgen.1007022)

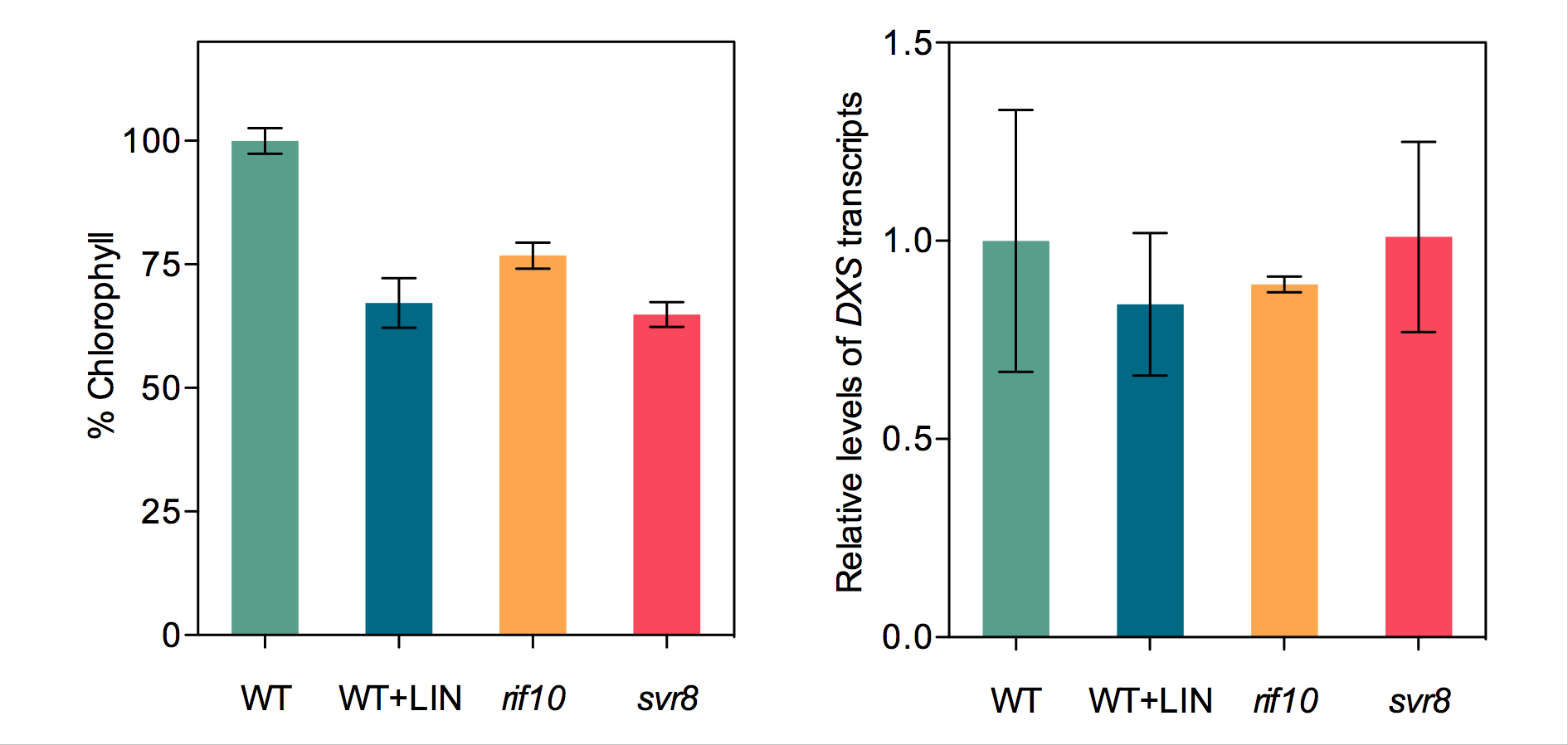

Supplement: S1 Fig — PGE was partially blocked either pharmacologically (by germinating and growing WT plants in the presence of 15μM LIN) or genetically (rif10-2 and svr8-2 mutants). Both WT+LIN and mutant plants showed a similar reduction in chlorophyll levels compared to untreated WT controls (left graph) but no differences in the levels of DXS transcripts (right graph). Chlorophyll quantification and DXS mRNA levels of 10-day-old WT, WT LIN-treated (15μM), rif10-2 and svr8-2. Data correspond to the mean and SEM values of n = 3 independent experiments. (TIFF) [file pgen.1007022.s001.tiff]

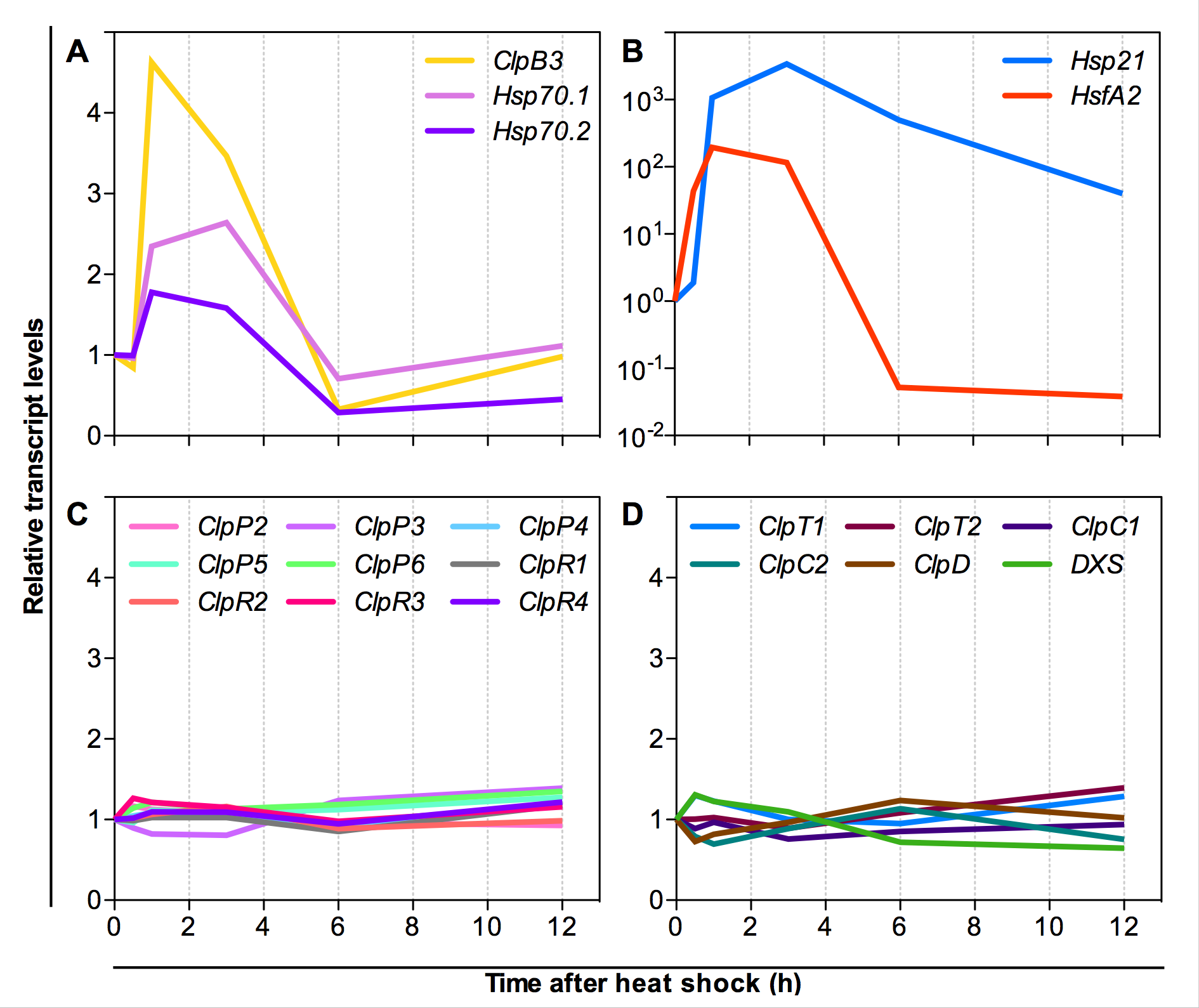

Supplement: S2 Fig — Data were obtained from the Arabidopsis eFP browser at www.bar.utoront.ca and correspond to the gene expression map of Arabidopsis abiotic (heat) stress treatment. Briefly, boxes with Arabidopsis plants grown under LD conditions on polypropylene rafts on MS medium supplemented with 0.5% agar and 0.5% sucrose were transferred from the growth chamber (at 24°C) to an incubator and exposed to a temperature of 38°C for 3h. Then, they were returned to the growth chamber and samples were collected at the indicated times. The results shown correspond to shoot samples. (TIFF) [file pgen.1007022.s002.tiff]

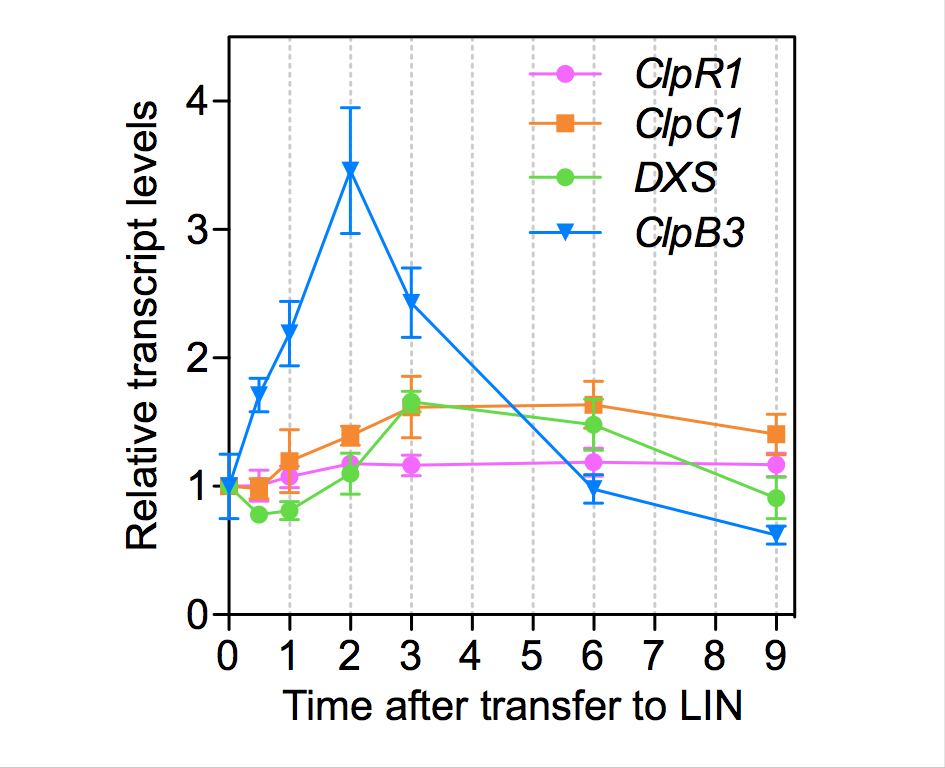

Supplement: S3 Fig — WT seedlings grown for 7 days on a mesh on top of MS solid medium were transferred to fresh medium containing 400 μM LIN and then whole-plant samples were collected at the indicated timepoints. Transcript levels are represented relative to untreated (0h) samples. Data correspond to the mean and SEM values of n≥3 independent experiments. (TIFF) [file pgen.1007022.s003.tiff]

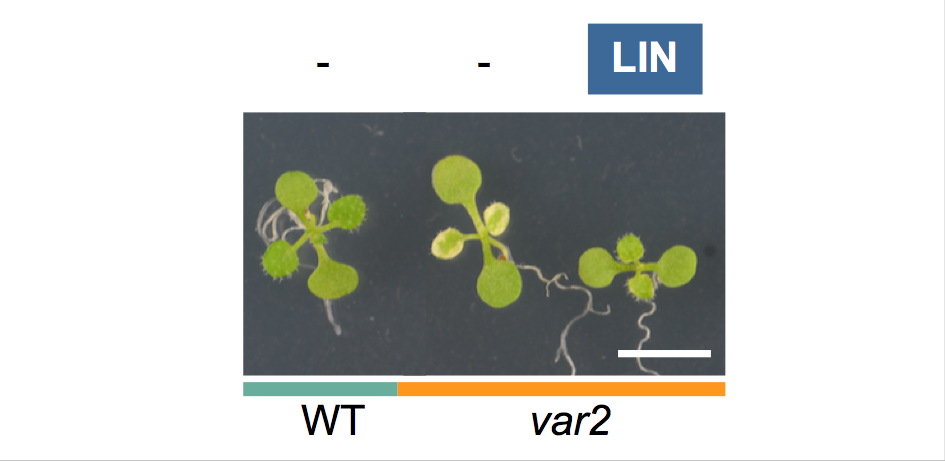

Supplement: S4 Fig — Picture shows representative individuals of WT and var2 plants germinated and grown for 10 days in the presence or absence of LIN (15μM). Bar, 5 mm. (TIFF) [file pgen.1007022.s004.tiff]

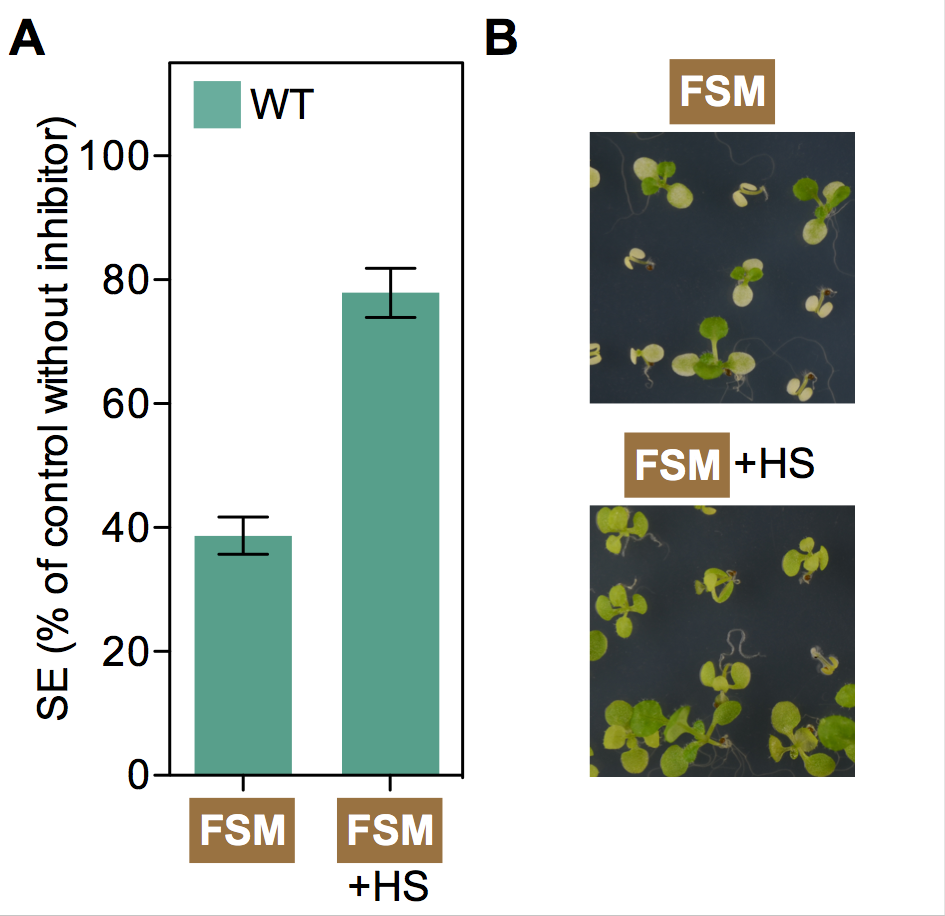

Supplement: S5 Fig — (A) Resistance was estimated by quantifying SE of 14-day-old WT seedlings germinated and grown at 22°C on plates of MS media supplemented with 30 μM FSM. Heat treatment was carried out by exposing the plates with the seedlings every day to 37°C for 90min. (B) Representative images of the seedlings. (TIFF) [file pgen.1007022.s005.tiff]

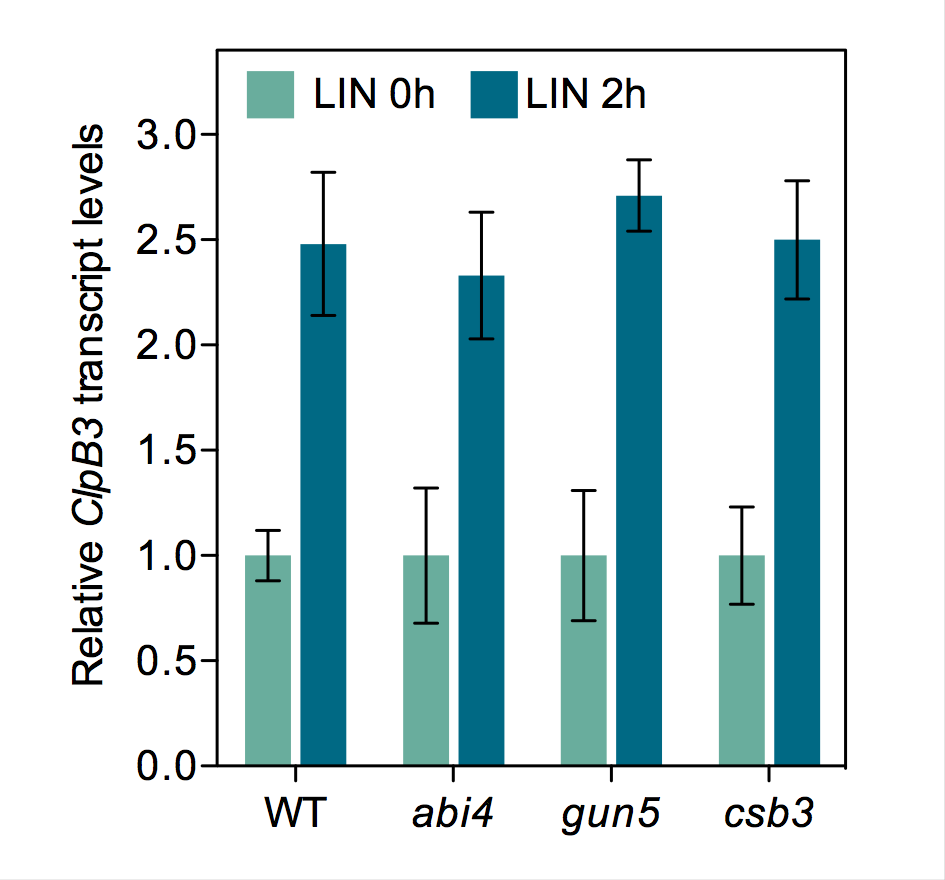

Supplement: S6 Fig — Transcript levels were quantified by qPCR analysis before and after transferring WT and mutant plants to medium with 400 μM LIN for 2h. Levels are represented relative to those in untreated (0h) controls. Data correspond to the mean and SEM values of n = 3 experiments. The csb3 mutant accumulates abnormally high levels of MEcPP. See Materials and methods for references on the mutants. (TIFF) [file pgen.1007022.s006.tiff]

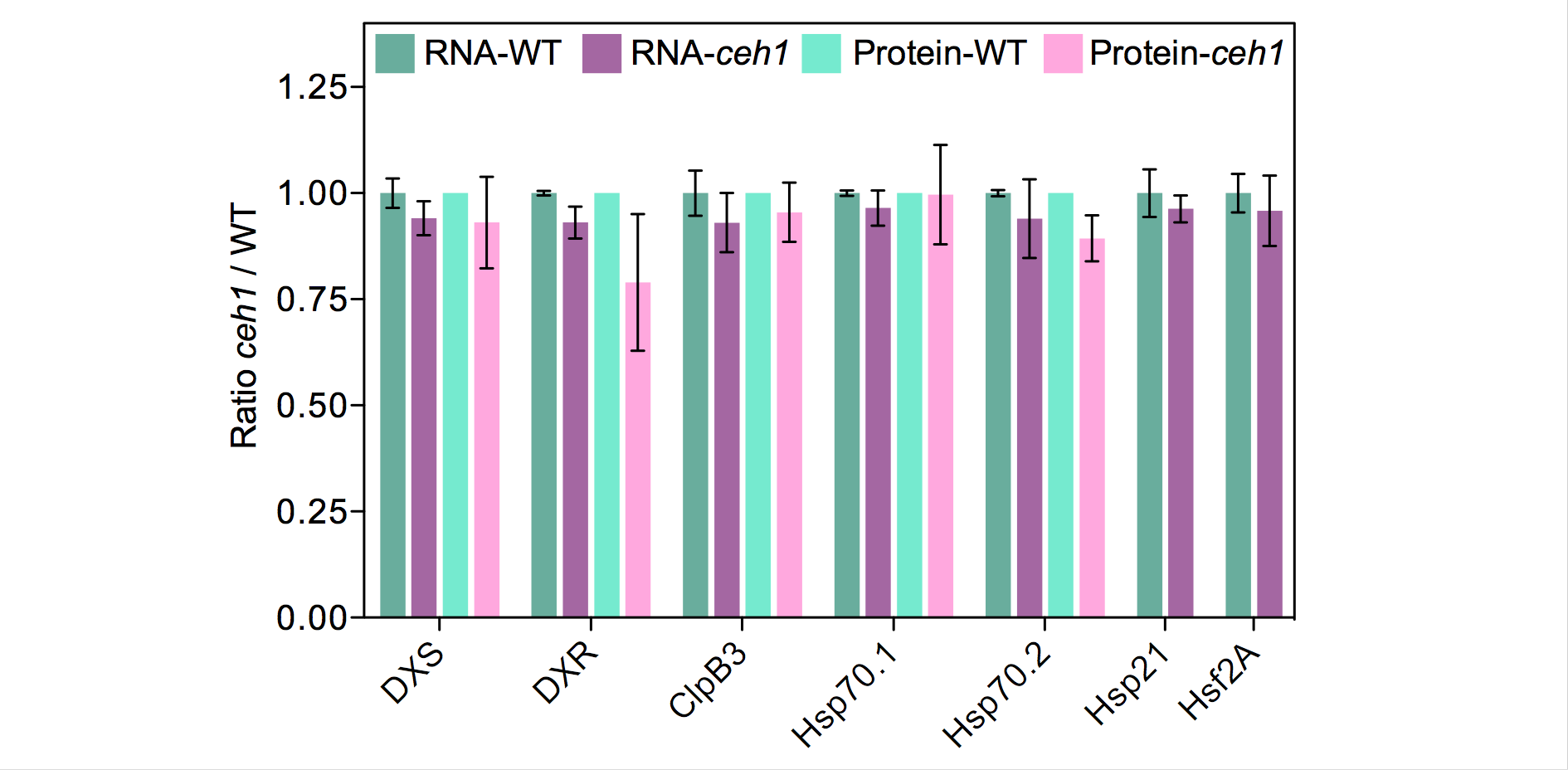

Supplement: S7 Fig — Levels are represented relative to those in WT plants. Data taken from Walley et al. (2015) Proc Natl Acad Sci USA 112: 6212–7, Supplemental Dataset S1. Hsp21 and HsfA2 proteins were not detected. (TIFF) [file pgen.1007022.s007.tiff]

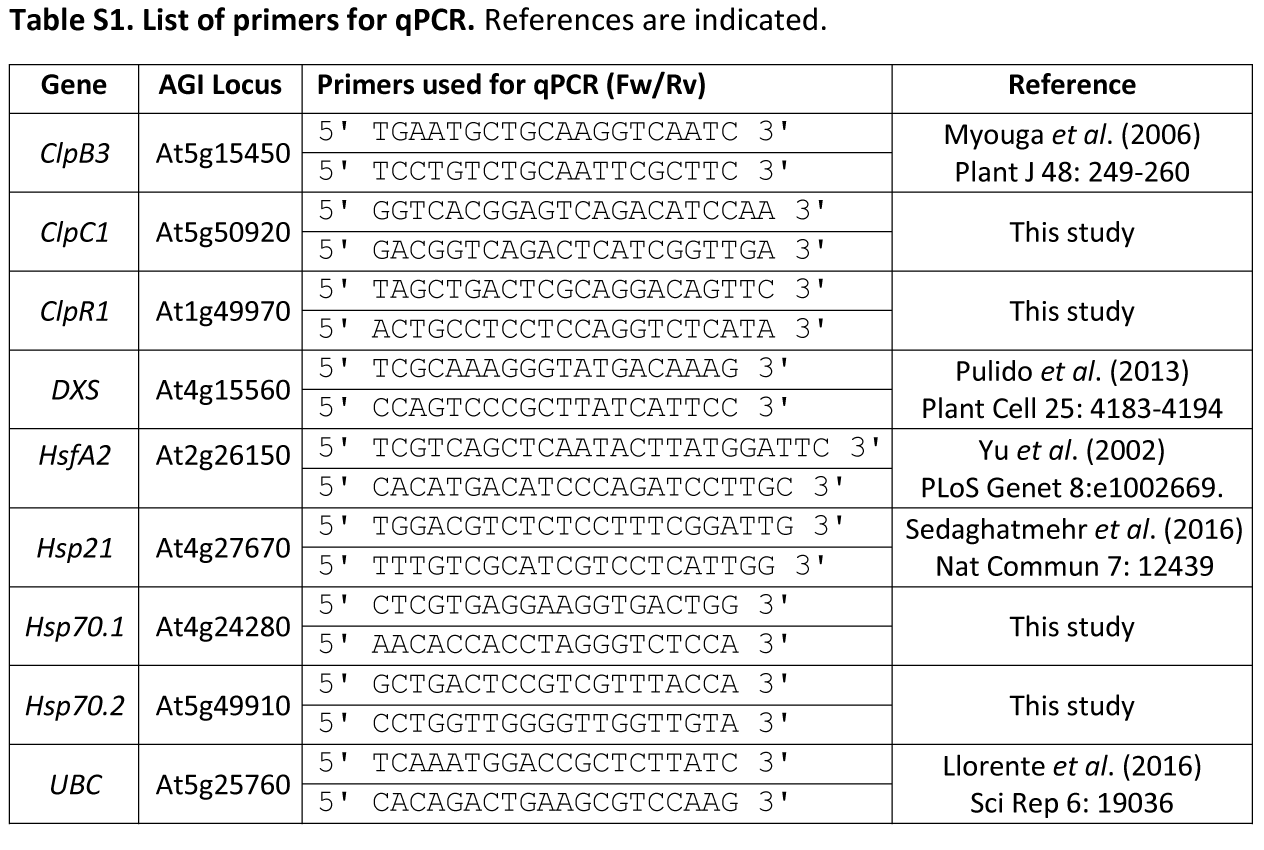

Supplement: S1 Table — References are indicated. (TIF) [file pgen.1007022.s008.tif]
